# The effect of the street environment on two types of essential physical activity in industrial neighborhoods from the perspective of public health: a study from the Harbin low-income population health survey, China

**DOI:** 10.1186/s12889-022-14533-7

**Published:** 2022-11-28

**Authors:** Yunjing Hou, Chaofan Zhai, Xiyu Chen, Wen Li

**Affiliations:** School of Landscape Architecture, Northeast Forestry University, 26 Hexing Road, Box 150040, Xiangfang District, Harbin City, Heilongjiang Province China

**Keywords:** Public health, Low-income people, Street environment, Physical activity, Industrial neighborhoods

## Abstract

A large number of low-income residents in industrial neighborhoods rarely engage in recreational and physical activities in green spaces in extremely cold weather. This study mainly explores the relationship between the street environment and physical activities under special industrial properties and extreme cold weather conditions. In addition, we further divide essential physical activity into two categories, life-type and traffic-type physical activity, to explore and refine the related studies.

We use principal component analysis to classify the street environment indicators and use multiple linear regression to analyze the impact of each indicator on different physical activities. The conclusions are as follows. For low-income people, the street environment in industrial neighborhoods has a much greater impact on life-type physical activity than traffic-type physical activity, and there is a conflict between the two. In addition, a high greening density is not conducive to either type of physical activity in the street environment. It reduces the paved area of streets and create sports conflicts between people undertaking different physical activities. The findings contribute to the development and optimization of public health research on environmental interventions in industrial neighborhood streets and enable effective recommendations for increasing outside physical activity among low-income people in severe weather conditions. In future studies, we will use the physical environment as a mediator to explore the relationship between the street environment and high-frequency chronic diseases in old industrial neighborhoods.

**Supplementary Information:**

The online version contains supplementary material available at 10.1186/s12889-022-14533-7.

## Introduction

The rise of China’s industrial cities mainly occurred in the 1950s, but with the rapid development of information technology in the twenty-first century, the industrial society gradually transitioned to an information society. The industrial city economy entered declined, and the industrial attributes were subsequently diluted. However, there are still many low-income groups, such as factory employees, their families and elderly retired workers, living in industrial neighborhoods, and they maintain their original behavioral habits and life patterns. Old industrial neighborhood green spaces often have difficulty meeting the physical and psychological needs of residents due to problems such as uneven spatial distributions and the poor quality of landscape vignettes.

Studies have found that increased access to green space is beneficial to residents’ physical and mental health [1]. However, among the various types of urban green spaces, existing studies emphasize the importance of streets. Streets are defined as road-based, linear, open spaces of various widths [2, 3].Streets can promote neighborhood health by providing safe entertainment and exercise spaces for residents to carry out physical activities [4, 5], which effectively reduces the negative impact of chronic diseases on residents’ mental health [6].Older people who live near streets with high-quality natural elements tend to participate in more outdoor activities on the street [7]. However, the current distribution of urban green spaces varies across neighborhoods with different types of residential populations. Affluent neighborhoods may enjoy a greater amount or higher quality of green space [8], while older neighborhoods with lower incomes tend to have less green space and to be of lower quality. In addition, the distance between neighborhoods and green spaces is particularly important for older people with mobility problems [9]. He et al. showed that street interventions had a positive impact on the walking time of residents living within two kilometers and that low-income groups benefited the most from street implementation in terms of walking time [10].

Notably, the built environment is defined as ‘the physical form of communities’, which can include land-use patterns, large- and small-scale built and natural features, and the transport system [11].Currently, the 3D model of the built environment proposed by Cervero and Koekelman [12] has been supplemented by Ewing and Cervero [13] to form the more widely used 5D model, which includes five dimensions: density, diversity, design, destination accessibility and distance to transit. As a result, many scholars, both in China and internationally, have conducted research on the elements of street environments that effectively promote the overall physical activity of residents. Barnett et al. explored the correlation between physical activity and street environments at three levels: walkability, infrastructure and safety. The study found that shop/commercial destinations, public transport, pedestrian-friendly infrastructure and the use of perceived safety measures were positively associated with overall physical activity and total walking [14]. He et al. conducted a study in Wuhan, China, and found that street greening was positively associated with more physical activity and that street connectivity and land use mix were also positively associated with the frequency of and total time spent in physical activity [15]. Chen et al. used pedestrian volume to represent the walking behavior at the population level, and the results showed that street greening, open sky, sidewalk and other microscale features effectively promoted pedestrian walking behavior [16].Stephanie et al. summarized 116 systematic reviews of high-income countries and found that there was a positive correlation between a positive walking environment (such as walking ability, walking infrastructure, street connectivity, land use combination) and adult/working age adults’ transportation physical activity [17]. In addition, Chang explored the relationship between outdoor activity and various urban street elements, and showed that participants who lived close to urban streets had higher levels of neighborhood social capital and better quality pathways, natural elements and seating and participated in outdoor activities more frequently [7]. Li et al. used the frequency and manner in which people use the street as a core reflection of street vibrancy and found that street width and transparency had a significant positive effect on street vibrancy [18].Several studies have shown that street greening has a positive impact on people’s sports activities [15, 16].

Recent relevant studies have also targeted specific types of people. A study from Iran explored the relationship between street network configuration in 18 primary schools and walking students. It found that the local morphological variables, integration and comprehensibility around the students’ homes had a direct and substantial relationship with the students’ walking, but the control and local selection indicators were negatively correlated. How children get to school has been found to be closely related to their physical and mental health [19].Ulrike et al. also investigated whether minor changes in the physical environment would increase the intensity of physical activity of individuals (especially children) in poor areas. Their research showed that street decoration led to spontaneous and creative positive play and social interaction between children and their peers or family members, which could help support support changes in physical activity behavior and social interaction [20]. Maria et al. also explored the impact of the urban environment on the physical activity of COPD patients. The study found that population density, pedestrian street length, slope and nitrogen dioxide exposure were related to the physical activity and ability of COPD patients living in densely populated areas [21]. However, the existing studies still have limitations in the selection of research fields and the classification of physical activity.

Scholars from different countries have also verified that the equity of green space use varies across income groups, with lower income residential areas within towns and cities likely to have the least access to green infrastructure such as green space or street trees [22–26]. Lower income groups also have less opportunity and time for leisure physical activities. Several recent studies have also confirmed that low-income ethnic minorities have unequal access to green spaces in terms of park size, quality and safety [27–33]. However, most studies have focused only on the frequency of leisure activities of residents in general urban neighborhoods, and few have focused on industrial neighborhoods, which have historical attributes and a higher proportion of low-income residents. These low-income neighborhoods often suffer more inequality and face more physical and mental health problems than the average neighborhoods [34]. This is because people who live in industrial neighborhoods face life stresses that prevent them from having the time and energy to engage in leisure activities, and they engage in only the necessary physical activities of daily life. Furthermore, previous experiments have examined only the relationship between physical activity as a whole and the street environment, however, the relationship between the two may vary depending on the type of physical activity [1].

Therefore, this paper investigates the health status and essential physical activity of low-income people in the street space of an industrial neighborhood in Harbin, an industrial city in China, during the cold season. On the one hand, it is obvious that the urban population density of China is much higher than that of most western countries, so there may be greater contradiction in the allocation of urban green resources. On the other hand, industrial cities often have a range of residential and service areas attached to factories, with a high degree of mixed land use and infrastructure centered on the factories. As a result, residents of industrial cities tend to meet their basic needs on foot and have a higher level of essential physical activity behavior than in other areas, which makes such cities likely to have higher street utilization. Most importantly, there are a large number of low-income groups in industrial communities, and their physical and mental health problems are often more serious than those in emerging urban communities. This research will help fill the gaps in the literature on the existing problems of industrial districts and promote the physical and mental health of low-income groups.

## Data and methods

### Research subjects

#### Characteristics of typical industrial neighborhoods and physical activity for low-income people

The study was conducted in Harbin (126°41′00.00″E, 45°45′00.00″N), a typical industrial city in China. At the end of the nineteenth century, the construction of the Middle East Railway enabled Harbin to develop rapidly from a small frontier fishing village into one of the major transportation hubs in Northeast Asia. The industrial structure of the city was gradually transformed from agriculture, animal husbandry and handicrafts to industry, and a large number of factories began to emerge in the center of the city. To meet the basic needs of factory workers, the old Harbin (now Xiangfang District) first started urban construction around the factories, and numerous industrial neighborhoods with factory unit compounds as the core were formed. However, with the shift in China’s economic development to coastal cities and the impact of new industries, Harbin’s industrial economy grew more slowly, and its industrial attributes declined, with many idle factories appearing in the city center becoming idle.

The streets in the industrial area considered in this study are old and mainly used for industrial transportation, and the roads are primarily connected by high-grade expressways connected to low-grade feeder roads. In addition, the neighborhood has a high percentage of low-income people (Table 1), who have no time for leisure physical activity their everyday life pressures. Physical activity for leisure and fitness purposes accounted for only 0.8% of the activity on our surveyed streets, so the study covers only two types of essential physical activity: life-type and traffic-type physical activity.

**Table 1:** Type and intensity of physical activity

Type of physical activity		Intensity of physical activity	
Life-type physical activity	Shopping	3–6 METs	Medium strength
	Dog walking	3–6 METs	
Traffic-type physical activity	Walking to and from work	3–6 METs	
	Waiting for the bus	1.5–3 METs	Low strength

Life-type physical activity, such as grocery shopping and dog walking, meets the needs of life, and there is a high level of neighborhood cohesion due to the presence of industrial plants. The neighborhood has a high degree of mixed land use and a wide variety of business types. The spatial structure of small neighborhoods and dense road networks also provides more options for daily travel for physically active crowds. Traffic-type physical activity, such as waiting for cars and other transportation commuting needs, meets the needs of daily work and study. The decay of the industrial plants has made it necessary for some residents to commute by public transportation, but some still walk to their jobs at the plants. According to the World Health Organization’s classification of physical activity intensity, both types of essential physical activity have MET values of less than 6, which is low to moderate intensity physical activity (see Table 1). In the high-stress living environment of the study area, low-to moderate-intensity physical activity provides additional opportunities to promote residents’ the physical and mental health.

#### Selection of typical industrial streets

**Fig 1:**
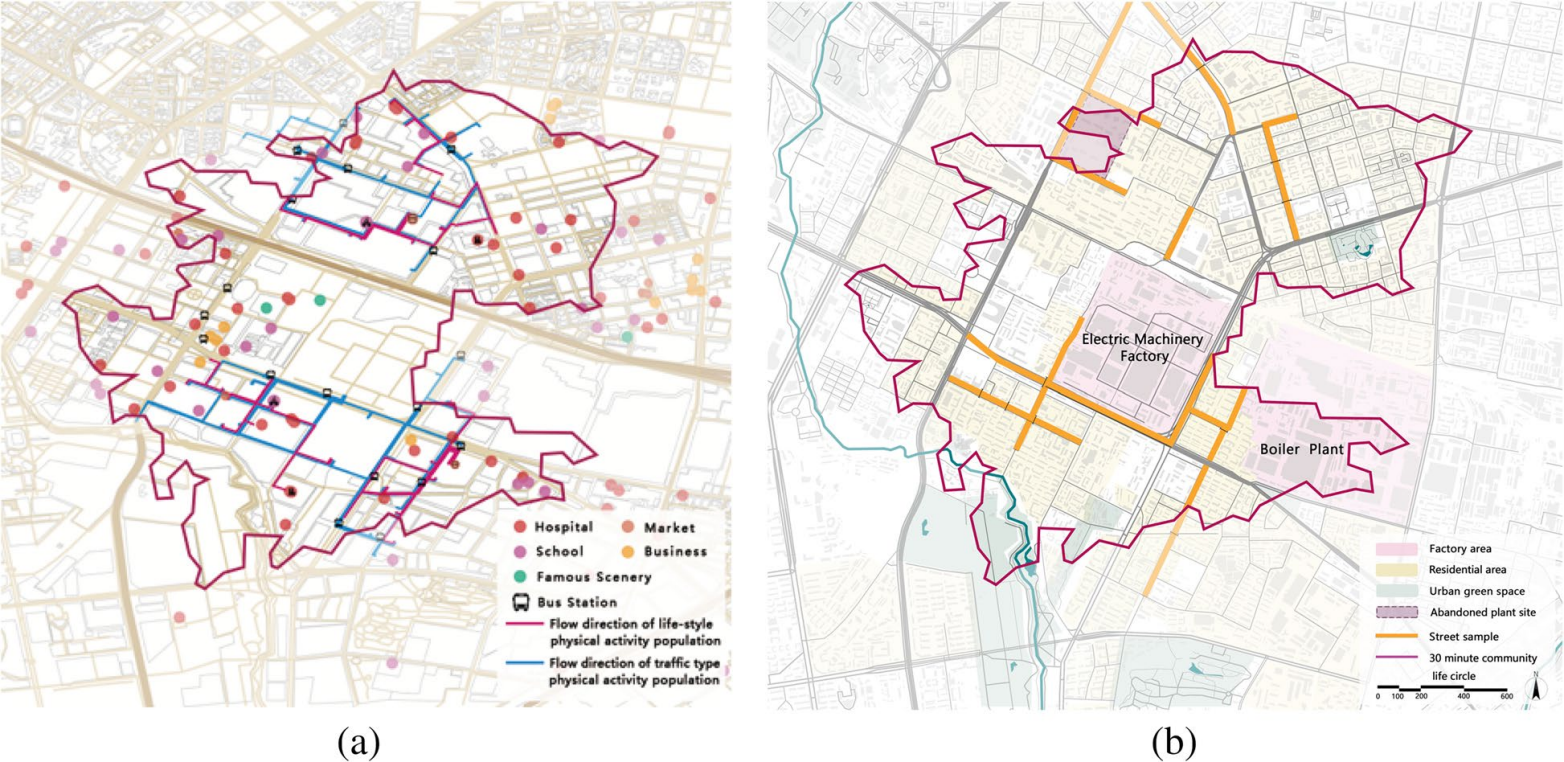
A sample of a typical industrial neighborhood street study. **a** Two types of essential physical activity footfall trajectories. **b** Study area street sample selection.

In this study, we took the existing Harbin Electric Factory as the center of a 30-minute living circle and selected 26 streets as the study sample (Fig. 1a). We investigated walking paths for the two types of essential physical activity in the sampled industrial area, prioritizing 20 streets with a high use frequency and selecting 6 low use frequency streets as a control sample (Fig. 1b). The streets were classified according to the Chinese Code for Urban Road Engineering Design (CJJ37–2012) classification standards, and the characteristics of the old industrial community streets themselves were divided into three classes: expressways, main roads and feeder roads. The three classes were selected equally for classification and included 3 expressways, 7 main roads and 16 feeder roads, of which few were affected by high grade expressways.

### Materials and methods

Assessing the relationship between the linear spatial street environment of industrial neighborhood streets and the two types of essential physical activity of low-income residents required the acquisition of data in 2 areas: street environment indicators and physical activity indicators. Both types of data were acquired by the researchers using objective measurements and subjective user evaluations to avoid overfocusing on objective indicators at the expense of the participants’ usage needs.

#### Street environment indicators

The first step is to obtain indicators of street environment characteristics, which are determined with reference to the 5D theory proposed by Ewing and Cervero [13]. These include the five dimensions of density, diversity, design, destination accessibility, and distance to transit, as shown in Table 2. The combination of big data and traditional methods can effectively eliminate the errors caused by a single method of acquisition.

**Table 2:** Quantitative approach to street environment indicators

Primary Index	Secondary Index		Quantitative Approach
Density	Density of life-type stores along the street (D1a)		(Number of life-type stores along the street/Average length of sidewalk on both sides) × 100
	Density of production-type street vendors (D1b)		(Number of production-type street vendors/Average length of sidewalk on both sides) × 100
	Density of production-type stores along the street (D1c)		(Number of production-type stores along the street/Average length of sidewalk on both sides) × 100
	Traffic density (D1d)		Streets are scored on a 5-point scale from very dense to very sparse based on the number of vehicles on the carriageway
	Parking density (D1e)		(Total number of parking on the left side/Length of sidewalk+Total number of parking on the right side/Length of sidewalk) × 100
	Greening density (D1f)		Vertical projection of trees, shrubs and ground cover/total area of stage walking space
Diversity	Diversity of commercial businesses along the street (D2)		$Entropy=-\sum_{i=1}^n{P}_i\cdot \ln {P}_i/\ln n$ ‘n’ denotes the number of business types involved in the study and ‘Pi’ denotes the proportion of size accounted for by the ith business type (the types of commercial space that are closely related to the daily travel of residents include:life-type stores along the street、traffic-type street vendors and traffic-type stores along the street)
Design	Average width of carriageway (D3a)		Average width of carriageway = Total area of carriageway/Total length of carriageway
	Number of road intersections(D3b)		Access via OSM Open Map
	Side interface height (D3c)		Acquired through Baidu Street View data
	Spatial organization characteristics (D3d-D3l)	Vehicle networks	Analysis of the axis model based on spatial syntax and includes several important indicators such as control value, connectivity value, choice, total integration and total depth value.
		Pedestrian networks	
Destination accessibility	Plant accessibility (DA1)		${L}_i=\frac{1}{n}\sum_{j=1}^n\mathit{\operatorname{Min}}\left({G}_j\right)$ Accessible distance based on the road network: the minimum distance method calculates the minimum distance to the destination, where‘L _i_’is the reachable distance to amenities on street i,‘j’is the jth road intersection on street i, ‘n’is the total number of road intersections on street i, and Min (G_j_) is the shortest distance from the jth intersection to a factory, neighborhood, or green space
	Community accessibility (DA2)		
	Green space accessibility (DA3)		
Distance to transit	Number of bus stations (DTT1)		Acquired from POI data
	Number of bus stops running routes (DTT2)		

**Fig. 2:**
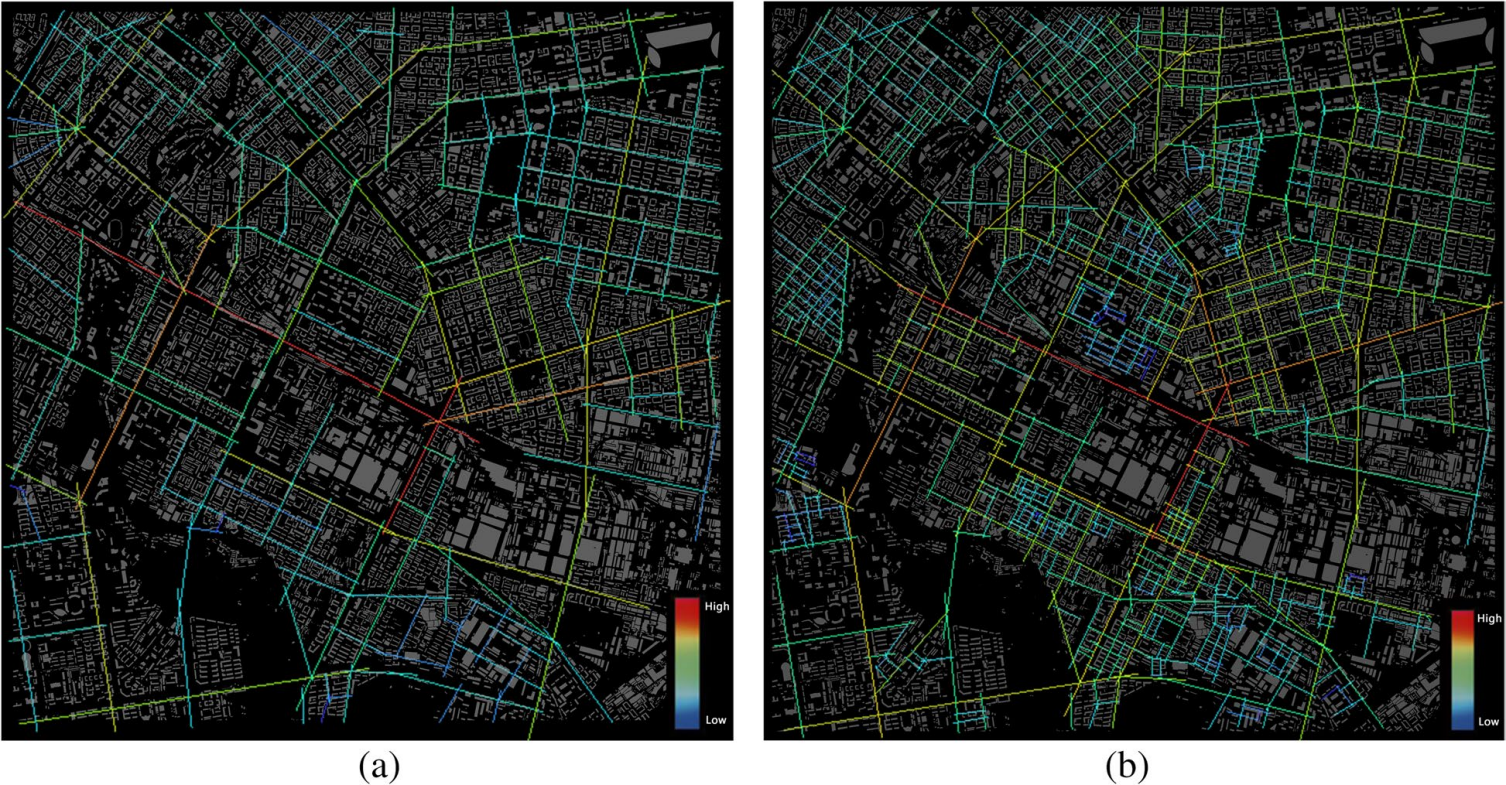
Classification of indicators of spatial organization characteristics. **a** Total integration (Vehicle networks). **b** Total integration (Pedestrian networks)

Among these, the spatial organization characteristic indicators refer to the analysis of the axis model based on spatial syntax and include several important indicators, such as control value, connectivity value, choice, total integration and total depth value. Depending on the vehicle and pedestrian trajectories, the author further classifies them into vehicle networks and pedestrian networks. The following figures shows the total integration of vehicle (Fig. 2a) and pedestrian perspectives (Fig. 2b). The vehicle network perspective examines the network of routes by which people travel by car or on foot through urban roads such as expressways, arterials and bypasses to the streets under study. The pedestrian network perspective examines internal roads in neighborhoods, neighborhood paths and open access roads that are difficult to access by motor vehicles in addition to the routes considered by the vehicle network perspective.

#### Physical activity indicators for low-income residents

1. Objective measurement

The total physical activity was measured from October to December 2021 at extremely cold temperatures (− 20 °C to –30 °C) with no snow and no clouds. The extreme season was chosen to maximize the exclusion of physical activity travel for leisure purposes. The data for each sample street included 5 weekdays and 2 weekend days for a total of 7 days, and the different types of physical activity were recorded in units of 0.5 h for each street for a total of 12 time points in the morning (7:30–9:00), midday (11:30–13:00) and evening (16:30–18:00). The researchers used cameras to spot video the crowds passing within the sample streets and automatically extracted the number of physically active people and artificially classified them through Baidu’s foot traffic statistics platform (https://cloud.baidu.com/product/body/num). Eventually, the total amount of physical activity in both categories was determined for different street segments and times.

(2) Self-reported mental health

Self-reported data were obtained through questionnaires and field visits, and 6–9 people were randomly selected from the streets of 26 sample streets to distribute questionnaires. The number of respondents in each street remained largely consistent, and a total of 215 valid questionnaires were collected. Among them, 180 valid questionnaires were collected from 20 high-frequency streets, and 35 valid questionnaires were collected from low-frequency streets due to the low number of users (see Table 3). The participants’ self-reported information included basic personal information and a self-assessment of health status. Basic personal information included the participants’ gender, age, marital status, living arrangement and monthly income. The self-evaluation of health status was based on the participants’ self-reported weekly time and frequency of essential physical activity, as well as their physical and mental health status. The two types of information were collected to explore the extent to which the objective street environment affected the residents’ mental health.

**Table 3:** Interviewees’ access to self reports

	High Frequency Street	Low Frequency Street
Average number of questionnaires collected in the street	9	6–7
Total number of valid questionnaires received	180	35

#### Statistical analyses and study procedure

All analyses in the paper were performed using IBM SPSS 25 software for data statistics. First of all, the collected self-reported data were analyzed by descriptive statistics to understand the usage of industrial streets. Then, Pearson correlation analysis was used to explore the correlation between street built environment indicators and two types of essential physical activities. After excluding the built environment indicators that have no correlation, we further identified the main components of the street environment index that affect different types of essential physical activities based on the principal component analysis. Then, we also explored the overall influence trend of street environment indicators on two types of essential physical activities through typical correlation analysis. Finally, a multiple regression model is established for each built environment indicator and two types of essential physical activities, and the importance of the built environment indicators is ranked to identify the main factors affecting the physical activity of the population.

## Results

### Health status report analysis of low-income residents in industrial neighborhoods

The respondents’ personal characteristics respondents are reported in Table 4. A total of 42.79% of the 215 respondents in the survey were male, and 57.21% were female; the respondents were mainly between 51 and 60 years old or over 60 years old, with 77.67% of the respondents being elderly; 76.74% of the respondents were married; the mode of residence was mainly three generations living together (38.14%) or living with a spouse (37.21%), indicating that the majority of older people chose to live with a partner or with their children, with fewer living alone. A total of 48.37% of the respondents had a monthly income of ¥2000 or less, which was lower than the average monthly income of ¥3334 in Harbin. In addition, the average monthly income of urban residents in Harbin was lower than the national average monthly income during the same period. This indicates that the overall monthly income level of residents within the study area was low, with a disproportionate number of older and low-income groups.

**Table 4:** Respondents’ basic personal information characteristics

Descriptive statistics of all participants (*N* = 215, Harbin, China in 2021)			
Variables	Item	Count	Percentage (%)
Individual-level variables			
Gender	Male	92	42.79
	Female	123	57.21
Age	18–35	10	4.65
	36–50	38	17.67
	51–60	94	43.72
	60 and above	73	33.95
Marital status	Single (Unmarried/Divorced/Widowed)	50	23.26
	Married	165	76.74
Living arrangement	Alone	19	8.84
	With spouse only	80	37.21
	Two generations together	34	15.81
	Three generations living under the same roof	82	38.14
Monthly income	Below ¥2000	104	48.37
	¥2000–4999	79	36.74
	¥5000–7999	19	8.84
	¥8000 and above	13	6.05
Physical health-level variables			
Physical activity time	<30 mins per week	64	29.77
	30–149 mins per week	100	46.51
	150–299 mins per week	27	12.56
	300–449 mins per week	11	5.12
	≥450 mins per week	13	6.05
Physical activity frequency	None	10	4.65
	Once a week	55	25.58
	2–4 times a week	78	36.28
	5–7 times a week	62	28.84
	More than 7 times a week	10	4.65
Physical health status	Very good	54	25.12
	Good	57	26.51
	Medium	53	24.65
	Not good	51	23.72
Depressed mood	None	8	3.72
	Occasionally	21	9.77
	Often	120	55.81
	Invariably	66	30.7
Chronic disease	Nonexistent	33	15.32
	Existence	182	84.65

According to the recommendations of the latest WHO guidelines on physical activity and sedentary behavior, in general, adults should undertake 150–300 minutes of moderate-intensity aerobic exercise or 75–150 minutes of higher-intensity aerobic exercise per week. Therefore, the questions I asked the respondents centered on their weekly physical activity and their personal health information. According to the survey, 76.28% of the respondents reported that they were physically active less than 150 minutes per week, and 29.77% were physically active less than 30 minutes per week, with only 23.73% of the participants in the study meeting the WHO guidelines. In terms of mental health, 86.51% of the participants often or always felt distressed and worried, indicating that residents of industrial areas may have more mental health problems. In terms of physical health, 48.37% of the respondents often felt physical pain, and 84.65% said they suffered from chronic diseases such as hypertension and diabetes.

### Correlation between street environment indicators and necessary physical activity

**Fig. 3:**
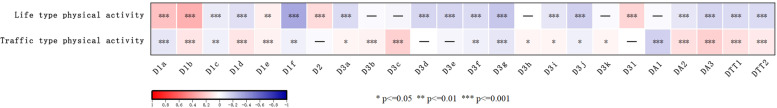
Pearson correlation analysis between the linear spatial street environment and the two types of necessary physical activity of the inhabitants of an industrial neighborhoods street

The independent variable is the linear spatial street environment indicators of the industrial neighborhood street, and the dependent variable is the two types of necessary physical activity among the residents. After measuring the above correlation data, Fig. 3 shows the results of the Pearson correlation between the two, and there are no significant correlations among the life-type physical activity and the number of road intersections, side interface height, connectivity value (vehicle networks) or control value (pedestrian networks). In addition, except for positive correlations with the density of life-type stores along the street, density of traffic-type street vendors, parking density, diversity of commercial businesses along the street and total depth value (pedestrian networks), life-type physical activity showed significant negative correlations with all other street environment indicators, with the strongest negative correlation of − 0.327 with greening density.

From Fig. 3, it can be concluded that there is no significant correlation between traffic-type physical activity and street environment indicators such as the diversity of commercial businesses along the street, control value (vehicle networks), connectivity value (vehicle networks) and total depth value (pedestrian networks). In contrast, traffic-type physical activity showed positive correlations with the main indicators of destination accessibility and distance to transit. In summary, there is a greater correlation between the convenience of reaching the destination and the traffic-type physically active crowd.

### Classification of street environment indicators of necessary physical activity

The different effects of each street environment indicator on the two types of necessary physical activity were further analyzed. After removing the street environment indicators that were not correlated in the bivariate correlation analysis, the main components of the street environment indicators that influenced the different essential types of physical activity were further clarified based on principal component analysis. In both principal component analyses, the KMO of the test sample was greater than 0.5, and the Sig of Bartlett’s spherical test was equal to 0.000 < 1%. Based on the comparison of the data, it can be concluded that the data in the paper are suitable for principal component analysis.

In the analysis of principal components, the street environment indicators were divided into three categories. Based on the commonality of the elements within the principal components, the three principal components affecting life-type physical activity were named travel accessibility (the eigenvalue components accounts for 44.094%), accessibility of living (the eigenvalue components accounts for 15.632%) and commercial richness (the eigenvalue components accounts for 10.868%); 70.594% of the total variance can be cumulatively explained. The three principal components affecting traffic-type physical activities were vehicle accessibility (the eigenvalue components accounts for 38.406%), pedestrian accessibility (the eigenvalue components accounts for 20.548%) and destinations available (the eigenvalue components accounts for 9.278%); 68.232% of the total variance can be explained accumulatively, as shown in Table 5. Pedestrianization has a significant effect on both necessary physical activities. Thus, humanized spatial organization is the main factor that influences the necessary physical activity of groups in the streets of industrial neighborhoods.

**Table 5:** Results of the classification of street environment indicators related to life-type physical activity

Life-type physical activity	Ingredient		
	A (Travel accessibility)	B (Accessibility of living facilities)	C (Commercial richness)
Total integration (Pedestrian networks) (D3k)	0.915		
Traffic density(D1d)	0.902		
Total depth value (Pedestrian networks) (D3l)	−0.898		
Average width of carriageway (D3a)	0.898		
Choice (Vehicle networks) (D3f)	0.857		
Choice (Pedestrian networks) (D3j)	0.822		
Number of bus stops running lines (DTT2)	0.813		
Total integration (Vehicle networks) (D3g)	0.801		
Parking density(D1e)	−0.695		
Number of bus stations (DTT1)	0.684		
Control value (Pedestrian networks) (D3h)	0.647		
Connectivity value (Vehicle networks) (D3e)	0.626		
Density of life-type stores along the street(D1a)	−0.569		
Green space accessibility (DA3)		−0.745	
Density of production-type stores along the street(D1c)		0.647	
Community accessibility (DA2)		−0.630	
Density of production-type street vendors(D1b)		0.596	
Diversity of commercial businesses along the street(D2)			−0.663
Greening density(D1f)			−0.659

**Fig. 4:**
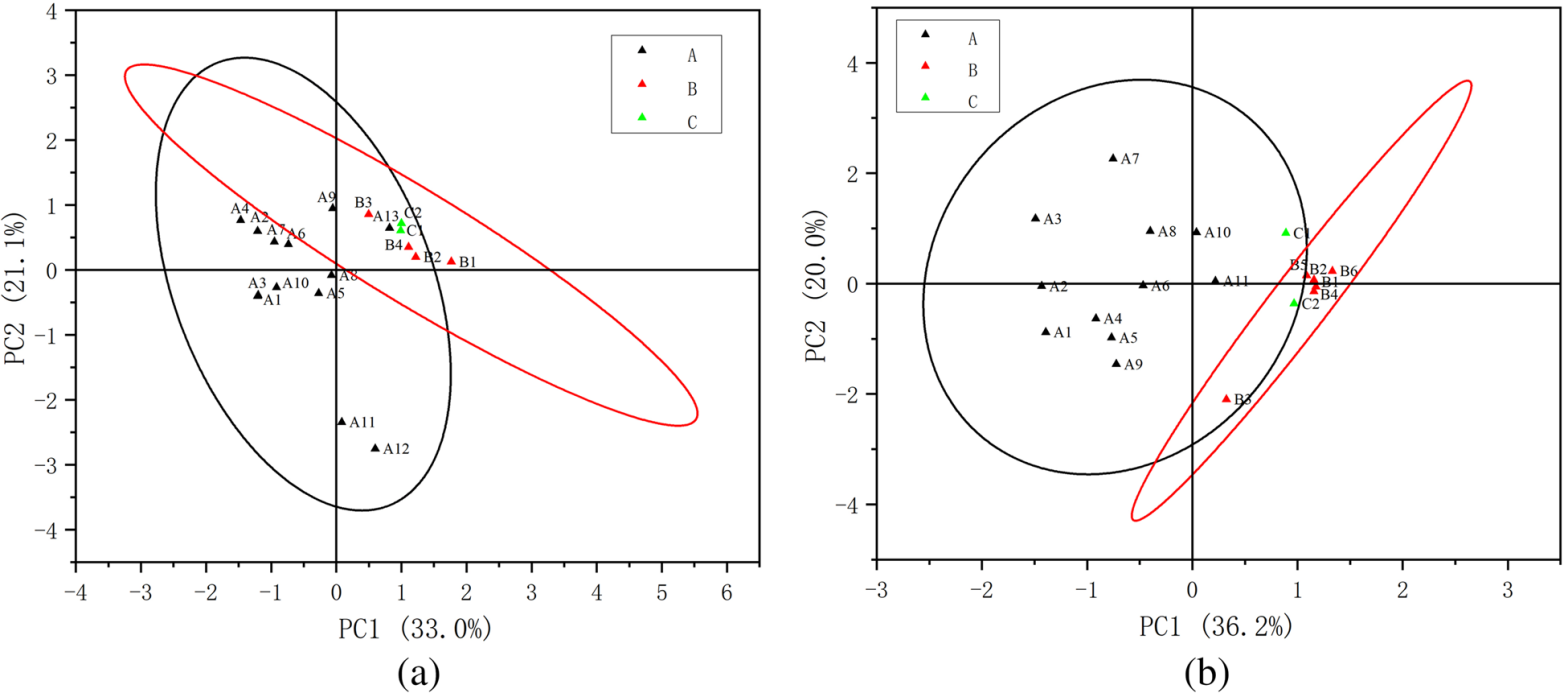
Results of principal component analysis. **a** Life-type physical activity confidence ellipse. **b** Traffic-type physical activity confidence ellipse

Within the study of essential physical activities, both types of activities are frequently influenced by accessibility. The accessibility of traffic-type physical activities can be categorized into vehicle accessibility, pedestrian accessibility and destinations available, as shown in Table 6. Industrial neighborhoods where transportation is the primary function are significantly influenced by vehicle accessibility. Factors such as the average width of the carriageway, traffic density and number of bus stops on active routes are based on public transportation considerations. The control value of pedestrian networks, connectivity value and number of road intersections are based on the convenience of pedestrian travel. In addition to the necessary travel accessibility, life-type physical activity is focused more on the accessibility of amenities and the richness of commerce (Fig. 4). At the same time, green space and community as necessary amenities for living have a significant impact on life-type physical activity.

**Table 6:** Results of the classification of street environment indicators related to traffic-type physical activity

Traffic-type physical activity	Ingredient		
	A (Vehicle Accessibility)	B (Pedestrian accessibility)	C (Destinations available)
Average width of carriageway(D3a)	0.920		
Traffic density(D1d)	0.919		
Total integration (Pedestrian networks) (D3k)	0.855		
Number of bus stops running lines (DTT2)	0.844		
Choice (Pedestrian networks) (D3j)	0.818		
Choice (Vehicle networks) (D3f)	0.813		
Total integration (Vehicle networks) (D3g)	0.747		
Parking density(D1e)	−0.721		
Number of bus stations (DTT1)	0.684		
Density of life-type stores along the street(D1a)	−0.638		
Community accessibility (DA2)	0.584		
Connectivity value (Pedestrian networks) (D3i)		0.822	
Control value (Pedestrian networks) (D3h)		0.817	
Number of road intersections(D3b)		0.706	
Density of production-type street vendors(D1b)		0.691	
Density of production-type stores along the street(D1c)		0.685	
Green space accessibility (DA3)		−0.564	
greening density(D1f)			− 0.564
Plant accessibility (DA1)			0.388

### Trends in the impact of the street environment indicators on the two types of necessary physical activities

**Fig. 5:**
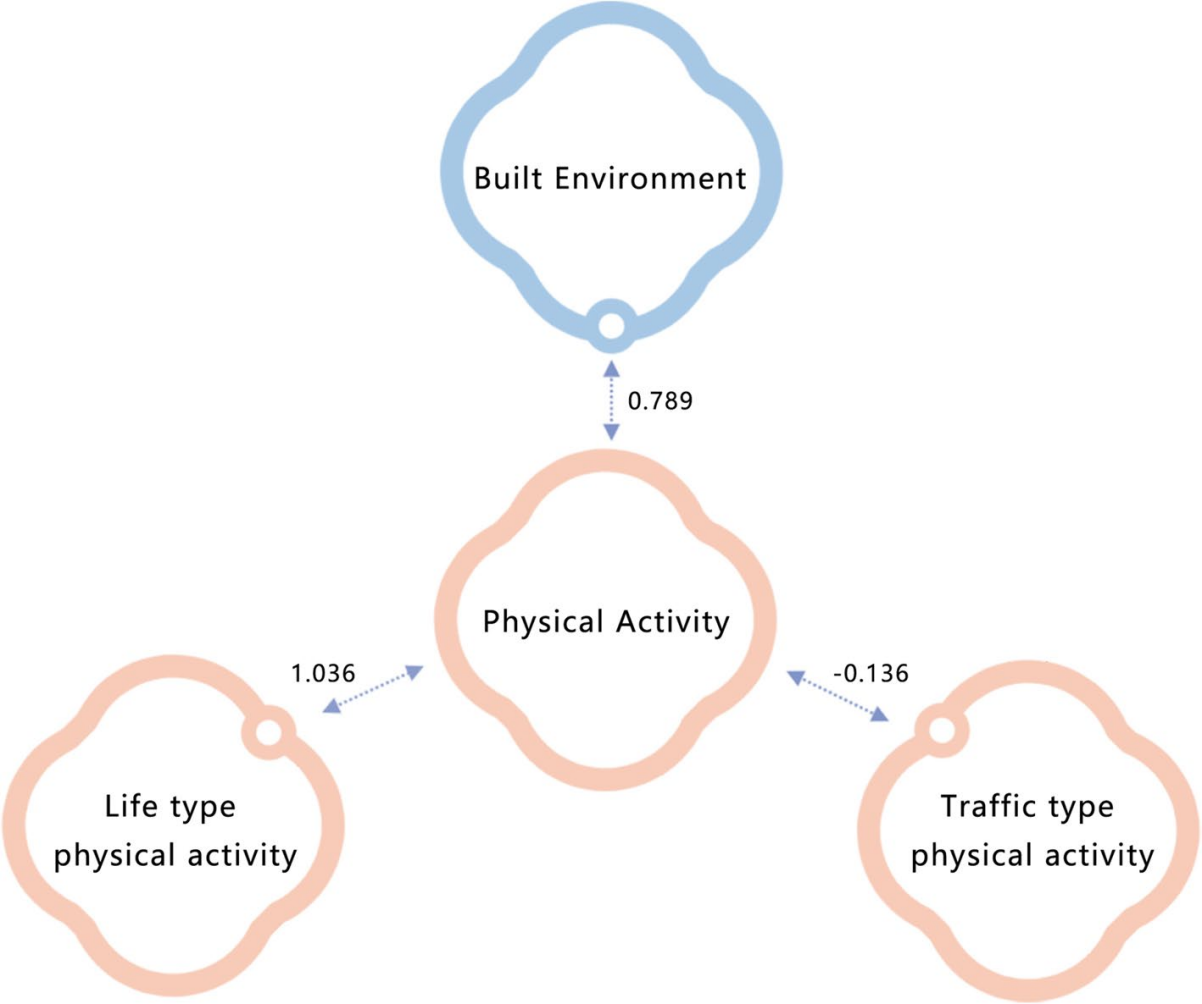
Results of a typical correlation analysis between street environment and two types of essential physical activity

Figure 5 shows the results of the typical correlation analysis. The street environment indicators in industrial neighborhoods showed a significant positive correlation with the two types of essential physical activity. At the same time, street environment indicators have a greater effect on the life-type physical activity of the two types of physical activities, with a correlation coefficient of 1.036. In addition, we also found contradictions between the two types of essential physical activities on the street, which indicates that the streets analyzed in the paper are not suitable for the two types of physical activity at the same time.

### Construction of regression models for the street environment indicators of necessary physical activity

In this paper, street environment indicators were used as independent variables, the number of people undertaking two types of essential physical activity during the research period was used as a dependent variable, and two multiple linear regression models were constructed using SPSS software. The coefficients of the influencing factors were ranked, and the top 5 street environment indicators in terms of degree of influence were extracted. The VIFs of the 2 models were < 10, and there was no significant covariance problem. The top 5 street environment indicators were ranked in descending order of importance: diversity of commercial businesses along the street, green space accessibility, number of bus stations, greening density and density of traffic-type stores along the street. Table 7 shows that all indicators were negatively correlated with each other, except for the diversity of commercial businesses along the street and the density of life-type stores along the street.

**Table 7:** Results of the construction of the life-type physical activity model

Type	Independent variable	Beta
Travel accessibility	Number of bus stations (D3b)	− 0.485^**^
Accessibility of living facilities	Green space accessibility (DA3)	− 0.564^**^
	Density of production-type stores along the street (D1c)	−0.304^**^
Commercial richness	Diversity of commercial businesses along the street (D2)	0.594^**^
	Greening density (D1f)	−0.468^**^

Table shows Pearson correlation coefficient values and significance values, *p < .05, **p < .01, ***p < .001, statistics significant at *p* < .05 are displayed in bold

The street environment indicators affecting traffic-type physical activity differed from those affecting life-type physical activity. Plant accessibility had the highest degree of influence, and neighborhood accessibility had the lowest degree of influence. The remaining three indicators were ranked in order of importance: the density of life-type stores along the street, greening density and number of road intersections. Among them, the number of road intersections and neighborhood accessibility were positively correlated. Table 8 shows that the accessibility of plants, the density of life-type stores and greening density were negatively correlated.

**Table 8:** Results of the construction of the traffic-type physical activity model

Type	Independent variable	Beta
Vehicle accessibility	Density of life-type stores along the street(D1a)	−0.687^**^
	Community accessibility (DA2)	0.112^*^
Pedestrian accessibility	Number of road intersections(D3b)	0.123^**^
Destinations available	Greening density(D2f)	−0.550^**^
	Plant accessibility (DA1)	−0.815^**^

Table shows Pearson correlation coefficient values and significance values, *p < .05, **p < .01, ***p < .001, statistics significant at *p* < .05 are displayed in bold

## Discussion

### The physiological state of the group with polarized income is worse, and the psychological state of the group of singles is worse

The demographic and sociological statistics of this study showed that Sig. > 0.05, indicating that the gender and age of the residents of industrial neighborhoods do not have a significant effect on the duration of their physical activity. This is consistent with the results of Vich et al. [35] In addition, the frequency of physical activity and the physical and mental health of the residents did not significantly differ with gender and age. Moreover, the duration and frequency of physical activity did not significantly differ with the residents’ monthly income, a result that differs from the findings of Jalón et al. that people with higher incomes are more likely to be physically active [1]. This difference is because the populations studied and residents of industrial neighborhoods rarely engage in physical activity on the streets. On the one hand, this may be due to the high motorization and dilapidated conditions of the streets in industrial neighborhoods, which do not support the daily leisure activities of their residents. On the other hand, the pressures of life may force residents to devote more time to work, which shortens their time for daily leisure physical activity. At the same time,the respondents may also have made appropriate adjustments when completing the self-assessment health questionnaire, and the true durations and frequencies of physical activity may be lower. Because the absence of linear green space largely reduces the probability of residents participating in outdoor leisure activities, a growing number of studies have also validated the relationship between street trees and positive physical activity outcomes [36–38]. Thus, increasing the linear green space of streets has a very important positive effect on increasing the probability of physical activity among residents of industrial neighborhoods.

Based on the data analysis, the physiological health status of the residents was significantly influenced by their monthly income, where the mean of 3.08 indicated that the residents with a monthly income of ¥8000 or more reported the worst physical health status. As indicated by the mean of 2.52, the residents with a monthly income below ¥2000 exhibited the second worst physical health status. This is different from the results of previous studies due to the specific characteristics of populations in industrial neighborhoods. Most residents in industrial neighborhoods are engaged in manual labor, primarily industrial labor, and higher pay means longer and more intense physical labor, thus increasing the chance of physical pain due to overwork. On the other hand, low-income residents may ignore the dangers of physical illnesses because of their economic constraints. This paper also shows that the mental health status of residents is affected by their marital status. Unmarried, divorced, and widowed residents show worse mental health than married residents, indicating that the married group can better communicate with those around them when psychological problems arise to alleviate mental health problems more efficiently.

### Mutual exclusion of life-type and traffic-type physical activities in the streets of industrial communities

The findings suggest that the effect of the street environment on physical activity in industrial neighborhoods varies with the type of physical activity. We followed the recommendations of previous related studies and classified the physical activities of the residents within the study area according to the purpose of use. This differs from previous studies, which often classified activities according to their intensity [1]. We found that life-type physical activity in industrial neighborhoods (1.036) was significantly more influenced by the street environment than traffic-type activity (− 0.136), further validating the conclusion that the influence of the built environment varies with physical activity purpose [39]. This suggests that the quality of the street environment in industrial neighborhoods has a more significant effect on physical activities such as grocery shopping and dog walking, as life-type physical activities tend to be more autonomous and selective. However, traffic-type physical activities often involve the use of the street based on the convenience of the destination, so it is likely that undertaking such an activity is not a resident’s active choice but rather the result of passive acceptance.

In addition, there is some conflict between the effects of the street environment on the two types of essential physical activity in the study area. In other words, characteristics of the street environment that are conducive to life-type physical activity may inhibit traffic-type physical activity. Therefore, the layout of the street environment in industrial areas should be effectively classified according to the physical activity needs of users, and the two types of physical activity paths should avoid overlapping. The hard and blind placement of street space elements does not necessarily play a positive role in promoting the behavioral needs of a neighborhood. Therefore, in the process of reconstructing old industrial streets, properly screening the ineffective space elements may create more efficient behavior paths for the two types of sports activity groups. This spatial environment will encourage the occurrence of physical activity behaviors in industrial neighborhoods, consciously distinguishe between the two types of behavioral path, guide the population to conduct physical activities in an orderly manner, and promote users’ physiological and psychological health levels in the process of engaging in physical activities.

### The variety of commercial formats along the street is more likely to help low income groups to meet multiple needs in their daily lives

The analysis in this paper finds that life-type physical activity in industrial neighborhoods is frequently influenced by commercial richness. A diversity of commercial businesses along the street is more likely to help people undertake life-type physical activity to meet multiple needs in their daily lives because low-income groups are more likely to choose to buy goods from affordable stores along the street than from commercially integrated groups. In terms of greening density, this paper also verifies the specificity of the low-income group. Contrary to the findings of previous studies, the density of trees exposed to the street and the daily walking time of individuals were positively correlated [15]. High greening density significantly reduces the number of life-type physical activities residents undertake in street spaces because of the inherent properties of older industrial neighborhoods, where transportation functions are predominant. High greening density reduces the hard surface area of streets, causing confusion in the passage of different types of physically active crowds and thereby substantially increasing time costs. This further confirms the conclusion in the previous subsection that there is a conflict between the two types of physical activities and clarifies that high green density is the main street space element that causes crowd conflict.

In summary, indicators such as enhanced greening density, greening ratio and greening coverage area do not meet the actual needs of users. Future design and renovation are needed based on the practicality of use. In addition, an increase in the density of traffic-type stores along the street and high green space accessibility also inhibit the life-type physical activities available to residents to some extent. The demand for traffic-type stores in older neighborhoods with declining industrial attributes is greatly reduced. Too many traffic-type stores along the streets reduces the number of life-type stores, which are in greater demand, and the number of people will be reduced as a result. Our recommendation is to consolidate the production-type stores along the street, so as to increase the number of life-type stores along the street, which are in greater demand. This effectively increases the chances that residents will leave their homes and maintain a high state of physical function. Streets with high green space accessibility tend to be perceived as having more people using them, but such green spaces are obviously more attractive than streets with less privacy and limited functions. Thus, streets with high green space accessibility tend to have a traffic-only function. This discovery will help the public to break the old impression and provide them with real street use in old industrial districts. During the study, the conclusion is that an increase in the number of bus stops will generate large numbers of people and lead to traffic flow confusion. It is recommended that separate public transport waiting areas be designated to ensure effective traffic flow. A reasonable street space layout can improve the efficiency of access for people engaged in essential physical activities, reduce the probability that the street space will be used less due to poor traffic, and enhance public walking opportunities.

### The high-density street built environment stimulates the occurrence of traffic conflicts for different physical activities

In the study of residents undertaking traffic-type physical activity, greening density showed the same results as for life-type physical activity. A high greening density is not conducive to various types of physical activities on streets. This differs from previous findings that high greenery promotes the occurrence of physical activity [15, 16]. This difference in findings is inextricably linked to the climatic environment and human characteristics of industrial neighborhoods. On the one hand, the long winter and short summer climate of Harbin makes users less dependent on street trees. The unique climatic conditions also limit the choice of greenery and the functionality of plants. On the other hand, residents of industrial districts are more concerned about the accessibility of the street than the shade and air purification functions of plants, and excessive greenery can impede residents’ street access. The different conclusions drawn based on specific geographical environments have enriched the theoretical research in the related field and provided the basis for subsequent studies.

**Fig. 6:**
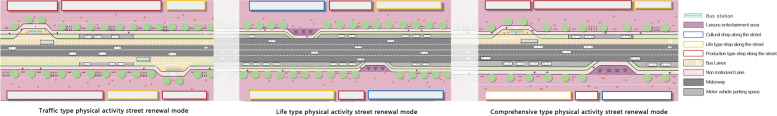
Forecast of street regeneration patterns in industrial neighborhoods

An increase in the density of life-type stores along the street and high plant accessibility will inhibit traffic-type physical activities because an increase in the density of life-type stores along the street increases the amount of life-type physical activity. The conflicting nature of the two types of activity was mentioned in the previous section, and an increase in such conflict would have a negative impact on traffic-type physical activity. Therefore, in the process of street renewal, a reduction in the number of life-type stores along such streets should be considered (Fig. 6). In the face of the decline in industrial properties, an increasing number of people undertaking traffic-type physical activities are moving from working locally to other commuting to work in other industries. In addition, an appropriate increase in the number of road intersections would provide more choices for physically active people engaged in traffic-type activities. In the future, we should advocate the urban planning model of “small blocks, dense road networks” to solve the traffic congestion problem caused by a single route and effectively improve the street traffic efficiency of physically active people.

## Conclusions

This study focuses on the effects of the street environment on essential physical activity (life-type and traffic-type physical activity) in the industrial neighborhoods of Harbin, an old industrial city in China. It was found that the high- and low-income groups showed worse physical health and that the married group had better mental health than the single group (including unmarried, divorced and widowed). This indicates that high- and low-income groups in industrial neighborhoods with lower overall income levels experience more life stress. Life-type physically active groups tend to be more active in peri-urban areas because the centers tend to have higher levels of consumption, while neighborhood residents prefer markets and fairs in nonurban centers. Traffic-type physical activity is strongly and positively correlated with the width of a carriageway, as wider carriageways facilitate the movement of vehicles. This helps meet the transportation needs of residents.

However, our study is limited in several ways. First, the streetscape data collection took place during the severe cold season, and the choice of season limited the availability of data on street vegetation after leaf fall (e.g., amount of vegetation, tree cover density, green space area and other green space indicators), which may have produced discrepancies with the results of the summer study. Second, the linear nature of the street itself may lead to bias when researchers obtain users’ physical activity data. Third, some of the reports in the study were based on subjects’ self-reported responses, and self-reported measures are subject to subjective choices that may be unreliable due to self-report bias. Finally, the relatively small sample size cannot fully reflect the street space use needs of the entire population in the industrial district. Nevertheless, self-reported data can still provide a wider range of responses and be beneficial in obtaining the views, perspectives and opinions of participants.

Therefore, according to the problems identified in the above article, we should consider the impact of seasons on data collection and increase the number of sample studies in future research to reduce the deviation of data caused by small sample size. In the selection of the study space, future studies should also consider broadening the type of green space studied; they should also consider including public green space and urban parks in comprehensive research experiments. At the same time, more efficient and objective measurement tools (e.g., wearable devices) should be explored for the acquisition of data on subjects’ conditions. In the future, we will try to explore the preference for green space in people suffering from high-frequency chronic diseases in China such as hypertension and diabetes. We hope to obtain green space configuration methods that can effectively alleviate residents’ chronic diseases and improve the physical and mental health of typical low-income residents in old industrial neighborhoods.

## Supplementary Information

**Additional file 1. **Street environment index data.

**Additional file 2. **Measurement of physical activity indicators of users.

**Additional file 3. **Supplementary data for data analysis.

## Data Availability

All data generated or analyzed during this study are included in this published article and its supplementary information files.
