# Supplementary material for: The effect of the street environment on two types of essential physical activity in industrial neighborhoods from the perspective of public health: a study from the Harbin low-income population health survey, China
Source: BMC Public Health. 2022 Nov 28;22:2201. doi: 10.1186/s12889-022-14533-7 (PMC9703667; doi:10.1186/s12889-022-14533-7)
Supplement: Supplementary file 1 — Additional file 1. Street environment index data. [file 12889_2022_14533_MOESM1_ESM.pdf]

Street environment index data							
Street sample	Density (D1)						Diversity (D2)
	D1a	D1b	D1c	D1d	D1e	D1f	D2
Street 1	2.40	0.00	0.10	5.00	1.30	25.37	0.20
Street 2	2.20	0.00	1.20	5.00	11.50	32.02	0.93
Street 3	5.00	0.40	0.00	4.00	0.00	29.78	0.38
Street 4	0.00	0.00	0.00	4.00	26.10	23.32	0.00
Street 5	1.10	0.00	1.90	1.00	46.70	8.30	0.96
Street 6	15.80	14.90	0.00	2.00	39.00	2.97	0.75
Street 7	10.08	0.53	0.00	2.00	63.52	9.19	0.29
Street 8	13.74	1.58	0.45	2.00	45.70	26.43	0.65
Street 9	5.88	0.46	0.31	5.00	0.09	20.37	0.42
Street 10	4.93	0.39	0.59	4.00	0.00	33.42	0.52
Street 11	7.51	0.59	0.44	4.00	22.69	9.71	0.41
Street 12	17.92	1.43	0.00	2.00	40.86	3.42	0.36
Street 13	24.49	0.40	6.41	2.00	65.03	3.37	0.21
Street 14	28.95	0.00	0.53	2.00	72.22	2.96	0.14
Street 15	18.72	0.00	1.28	2.00	66.84	2.43	0.33
Street 16	12.69	1.01	6.04	2.00	57.49	22.03	0.76
Street 17	23.79	2.41	1.72	2.00	59.26	6.54	0.48
Street 18	5.92	0.00	1.58	1.00	27.66	28.32	0.75
Street 19	6.29	1.08	0.18	2.00	35.49	38.03	0.46
Street 20	12.66	0.00	0.44	2.00	48.03	45.97	0.19
Street 21	3.43	0.95	0.38	3.00	40.71	35.18	0.53
Street 22	6.35	0.00	0.55	3.00	38.40	12.00	0.40
Street 23	17.84	5.39	2.49	2.00	37.53	39.45	0.75
Street 24	3.58	0.36	0.35	2.00	73.12	52.80	0.50
Street 25	12.43	0.78	0.39	2.00	68.95	53.39	0.34
Street 26	22.67	0.00	0.44	3.00	7.73	0.82	0.14
Street sample	Design (D3)						
	D3a	D3b	D3c	D3d	D3e	D3f	D3g
Street 1	58.40	4.00	22.00	2.45	7.00	3107.00	1.77
Street 2	60.00	2.00	14.40	1.73	8.00	6307.00	2.29
Street 3	44.00	3.00	25.10	0.70	4.00	537.00	1.58
Street 4	35.00	3.00	51.00	0.79	3.00	488.00	1.60
Street 5	17.50	2.00	15.40	0.14	0.00	0.00	1.29
Street 6	17.00	3.00	14.40	0.59	3.00	58.00	1.31
Street 7	21.00	4.00	43.20	1.20	4.00	18.00	1.10
Street 8	18.00	5.00	53.30	1.29	6.00	1230.00	1.55
Street 9	58.40	3.00	11.51	2.45	7.00	3107.00	1.77
Street 10	44.00	3.00	17.69	0.70	4.00	918.00	1.58
Street 11	35.00	3.00	19.62	0.79	3.00	356.00	1.60
Street 12	15.00	2.00	28.86	0.59	3.00	58.00	1.31
Street 13	21.00	3.00	35.40	1.20	4.00	18.00	1.10
Street 14	21.00	3.00	23.44	1.20	4.00	18.00	1.10
Street 15	21.00	3.00	18.88	1.20	4.00	18.00	1.10
Street 16	18.00	4.00	18.53	1.29	6.00	1230.00	1.55
Street 17	15.00	3.00	20.37	1.29	6.00	64.00	1.55
Street 18	9.00	2.00	20.06	1.29	6.00	64.00	1.55
Street 19	10.00	2.00	57.77	0.83	2.00	2.00	1.21

Street 20	8.00	2.00	20.13	0.46	2.00	138.00	1.56
Street 21	26.00	2.00	58.03	1.62	5.00	377.00	1.44
Street 22	33.00	2.00	15.48	0.99	4.00	710.00	1.70
Street 23	9.00	3.00	18.54	0.81	5.00	477.00	1.67
Street 24	9.00	3.00	17.81	0.81	5.00	477.00	1.67
Street 25	9.00	2.00	25.42	0.81	5.00	477.00	1.67
Street 26	20.00	2.00	21.00	1.12	5.00	369.00	1.58

Street sample	Design (D3)				
	D3h	D3i	D3j	D3k	D3l
Street 1	10.00	31995.00	1.91	2.61	2458.00
Street 2	11.00	87716.00	2.22	2.36	2192.00
Street 3	10.00	27112.00	1.77	2.14	2610.00
Street 4	6.00	25615.00	1.76	1.37	2621.00
Street 5	2.00	407.00	1.49	0.43	3004.00
Street 6	9.00	11343.00	1.53	2.05	2932.00
Street 7	15.00	12947.00	1.46	4.33	3052.00
Street 8	20.00	24528.00	1.75	6.62	2639.00
Street 9	10.00	31995.00	1.91	2.61	2458.00
Street 10	10.00	29424.00	1.77	2.14	2610.00
Street 11	6.00	17853.00	1.76	1.37	2621.00
Street 12	9.00	11343.00	1.53	2.05	2932.00
Street 13	15.00	12947.00	1.46	4.33	3052.00
Street 14	15.00	12947.00	1.46	4.33	3052.00
Street 15	15.00	12947.00	1.46	4.33	3052.00
Street 16	20.00	24528.00	1.75	6.62	2639.00
Street 17	20.00	1012.00	1.75	6.62	2639.00
Street 18	20.00	1012.00	1.75	6.62	2639.00
Street 19	5.00	8700.00	1.36	1.11	3131.00
Street 20	5.00	2089.00	1.67	1.07	2732.00
Street 21	17.00	25278.00	1.66	5.03	2751.00
Street 22	8.00	23659.00	1.84	1.65	2531.00
Street 23	10.00	1919.00	1.56	2.77	2879.00
Street 24	10.00	1919.00	1.56	2.77	2879.00
Street 25	10.00	1919.00	1.56	2.77	2879.00
Street 26	8.00	4126.00	1.73	1.50	2660.00

Street sample	Destination accessibility (DA)			Distance to transit (DTT)	
	DA1	DA2	DA3	DTT1	DTT2
Street 1	831.25	88.25	1322.00	2.00	20.00
Street 2	333.25	55.00	478.50	2.00	16.00
Street 3	1268.50	192.50	1092.50	1.00	9.00
Street 4	351.67	81.00	2392.67	1.00	16.00
Street 5	668.50	79.50	958.00	0.00	0.00
Street 6	690.00	48.33	427.33	0.00	0.00
Street 7	537.25	43.50	568.50	2.00	4.00
Street 8	435.20	42.00	418.00	4.00	10.00
Street 9	322.00	114.00	480.33	3.00	39.00
Street 10	1458.33	86.67	1150.33	1.00	12.00
Street 11	639.67	130.33	1937.67	2.00	19.00
Street 12	901.50	56.50	295.00	0.00	0.00

Street 13	536.33	51.67	583.33	1.00	2.00
Street 14	1034.33	60.00	719.33	1.00	3.00
Street 15	1243.00	55.00	566.33	0.00	0.00
Street 16	587.50	61.00	408.00	2.00	6.00
Street 17	692.00	40.00	581.00	0.00	0.00
Street 18	586.00	41.30	802.00	0.00	0.00
Street 19	512.50	46.00	2425.00	0.00	0.00
Street 20	332.00	47.50	508.00	0.00	0.00
Street 21	697.00	99.50	1852.50	1.00	8.00
Street 22	1335.00	93.50	1442.50	1.00	7.00
Street 23	1972.67	34.00	968.00	0.00	0.00
Street 24	1739.00	39.00	675.67	0.00	0.00
Street 25	1462.00	54.00	408.50	0.00	0.00
Street 26	1963.00	38.50	860.50	1.00	2.00

Note:The determination of street built environment indicators refers to the 5D theory proposed by Ewing & Cervero (2010) and the field investigation of participants, including five dimensions: density, diversity, design, destination accessibility and distance to transit.
See Table 1 in the text for specific quantification methods
