# Supplementary material for: The effect of the street environment on two types of essential physical activity in industrial neighborhoods from the perspective of public health: a study from the Harbin low-income population health survey, China
Source: BMC Public Health. 2022 Nov 28;22:2201. doi: 10.1186/s12889-022-14533-7 (PMC9703667; doi:10.1186/s12889-022-14533-7)

Measurement of physical activity indicators of users				
Time: October to December 2021		Weather: no snow and no clouds		
Time		Street sample	life-type physical activity	traffic-type physical activity
Day 1	7:30	Street 1	4	232
		Street 2	1	72
		Street 3	1	119
		Street 4	2	212
		Street 5	8	41
		Street 6	46	194
		Street 7	23	437
		Street 8	8	115
		Street 9	11	168
		Street 10	0	178
		Street 11	8	227
		Street 12	31	129
		Street 13	13	69
		Street 14	9	55
		Street 15	12	51
		Street 16	13	172
		Street 17	15	124
		Street 18	1	214
		Street 19	8	231
		Street 20	2	87
		Street 21	53	375
		Street 22	11	67
		Street 23	2	19
		Street 24	5	337
		Street 25	1	141
		Street 26	3	31
	8:00	Street 1	3	77
		Street 2	1	34
		Street 3	2	50
		Street 4	0	105
		Street 5	2	43
		Street 6	37	178
		Street 7	15	143
		Street 8	11	96
		Street 9	13	61
		Street 10	1	74
		Street 11	10	369
		Street 12	25	119
		Street 13	8	41
		Street 14	6	36
		Street 15	2	22
		Street 16	16	142
		Street 17	12	57
		Street 18	0	45
		Street 19	3	68

		Street 20	4	36
		Street 21	24	151
		Street 22	2	27
		Street 23	4	9
		Street 24	3	101
		Street 25	3	36
		Street 26	1	10
	8:30	Street 1	4	79
		Street 2	3	36
		Street 3	3	46
		Street 4	2	59
		Street 5	4	20
		Street 6	70	145
		Street 7	15	148
		Street 8	14	106
		Street 9	9	59
		Street 10	1	70
		Street 11	14	89
		Street 12	47	96
		Street 13	8	39
		Street 14	5	21
		Street 15	2	20
		Street 16	20	159
		Street 17	5	71
		Street 18	1	28
		Street 19	10	82
		Street 20	1	21
		Street 21	35	71
		Street 22	0	35
		Street 23	2	24
		Street 24	2	51
		Street 25	1	22
		Street 26	1	14
	9:00	Street 1	6	58
		Street 2	5	41
		Street 3	4	34
		Street 4	3	55
		Street 5	5	17
		Street 6	48	103
		Street 7	7	69
		Street 8	22	128
		Street 9	8	26
		Street 10	3	50
		Street 11	9	87
		Street 12	31	68
		Street 13	5	24
		Street 14	4	16
		Street 15	4	12
		Street 16	15	191
		Street 17	9	63
		Street 18	1	21

		Street 19	1	34
		Street 20	0	22
		Street 21	1	41
		Street 22	0	27
		Street 23	4	35
		Street 24	1	55
		Street 25	6	44
		Street 26	0	11
	11:30	Street 1	3	23
		Street 2	2	33
		Street 3	2	31
		Street 4	2	47
		Street 5	1	7
		Street 6	29	52
		Street 7	20	112
		Street 8	10	78
		Street 9	6	12
		Street 10	2	46
		Street 11	10	253
		Street 12	19	35
		Street 13	8	39
		Street 14	6	28
		Street 15	3	15
		Street 16	15	52
		Street 17	19	59
		Street 18	0	147
		Street 19	8	38
		Street 20	3	27
		Street 21	12	409
		Street 22	3	45
		Street 23	9	62
		Street 24	1	147
		Street 25	5	52
		Street 26	1	17
	12:00	Street 1	1	51
		Street 2	3	24
		Street 3	3	48
		Street 4	7	50
		Street 5	2	18
		Street 6	37	75
		Street 7	7	67
		Street 8	9	46
		Street 9	8	31
		Street 10	0	70
		Street 11	16	386
		Street 12	24	49
		Street 13	2	22
		Street 14	1	18
		Street 15	1	12
		Street 16	15	69
		Street 17	10	43

		Street 18	0	30
		Street 19	7	42
		Street 20	5	12
		Street 21	5	198
		Street 22	4	40
		Street 23	7	35
		Street 24	0	102
		Street 25	2	54
		Street 26	4	18
	12:30	Street 1	2	20
		Street 2	2	17
		Street 3	4	39
		Street 4	7	96
		Street 5	2	14
		Street 6	44	67
		Street 7	13	65
		Street 8	10	41
		Street 9	7	10
		Street 10	5	57
		Street 11	33	250
		Street 12	29	44
		Street 13	4	17
		Street 14	3	14
		Street 15	2	10
		Street 16	14	62
		Street 17	10	77
		Street 18	2	10
		Street 19	3	17
		Street 20	2	24
		Street 21	8	225
		Street 22	1	41
		Street 23	7	26
		Street 24	3	50
		Street 25	1	15
		Street 26	0	21
	13:00	Street 1	3	42
		Street 2	1	27
		Street 3	2	50
		Street 4	4	79
		Street 5	4	11
		Street 6	52	75
		Street 7	8	51
		Street 8	8	35
		Street 9	11	36
		Street 10	3	76
		Street 11	8	147
		Street 12	35	49
		Street 13	2	15
		Street 14	0	12
		Street 15	2	9
		Street 16	12	53

		Street 17	18	43
		Street 18	2	27
		Street 19	10	31
		Street 20	0	21
		Street 21	9	119
		Street 22	4	28
		Street 23	7	39
		Street 24	2	44
		Street 25	0	19
		Street 26	3	19
	16:30	Street 1	3	42
		Street 2	1	27
		Street 3	2	51
		Street 4	4	79
		Street 5	3	24
		Street 6	106	99
		Street 7	8	103
		Street 8	19	55
		Street 9	12	22
		Street 10	1	76
		Street 11	15	89
		Street 12	71	65
		Street 13	3	21
		Street 14	1	16
		Street 15	1	11
		Street 16	29	83
		Street 17	57	72
		Street 18	0	16
		Street 19	2	26
		Street 20	2	16
		Street 21	24	233
		Street 22	4	24
		Street 23	2	8
		Street 24	1	46
		Street 25	2	20
		Street 26	1	12
	17:00	Street 1	9	144
		Street 2	1	78
		Street 3	2	54
		Street 4	4	52
		Street 5	3	19
		Street 6	128	122
		Street 7	8	142
		Street 8	21	106
		Street 9	15	97
		Street 10	2	80
		Street 11	4	236
		Street 12	102	81
		Street 13	9	26
		Street 14	6	23
		Street 15	5	10

		Street 16	31	158
		Street 17	13	50
		Street 18	22	19
		Street 19	9	71
		Street 20	4	21
		Street 21	45	273
		Street 22	8	36
		Street 23	4	31
		Street 24	12	408
		Street 25	5	6
		Street 26	3	3
	17:30	Street 1	8	96
		Street 2	3	30
		Street 3	1	70
		Street 4	2	72
		Street 5	6	23
		Street 6	100	167
		Street 7	26	297
		Street 8	25	52
		Street 9	19	69
		Street 10	0	105
		Street 11	11	277
		Street 12	78	112
		Street 13	12	47
		Street 14	5	29
		Street 15	2	21
		Street 16	39	78
		Street 17	23	51
		Street 18	30	18
		Street 19	27	49
		Street 20	15	43
		Street 21	2	187
		Street 22	4	44
		Street 23	2	35
		Street 24	3	145
		Street 25	4	36
		Street 26	2	15
	18:00	Street 1	2	49
		Street 2	2	58
		Street 3	3	32
		Street 4	3	102
		Street 5	7	14
		Street 6	79	118
		Street 7	12	92
		Street 8	11	44
		Street 9	6	33
		Street 10	3	46
		Street 11	9	515
		Street 12	52	79
		Street 13	2	26
		Street 14	1	16

		Street 15	1	11
		Street 16	8	67
		Street 17	4	65
		Street 18	6	10
		Street 19	5	115
		Street 20	2	17
		Street 21	11	133
		Street 22	3	35
		Street 23	5	16
		Street 24	3	23
		Street 25	2	3
		Street 26	1	29
Day 2		Street 1	8	294
		Street 2	2	68
		Street 3	2	130
		Street 4	4	194
		Street 5	14	47
		Street 6	53	258
		Street 7	21	494
		Street 8	10	108
		Street 9	16	187
		Street 10	1	195
		Street 11	10	207
		Street 12	35	172
	7:30	Street 13	9	102
		Street 14	5	69
		Street 15	5	63
		Street 16	15	161
		Street 17	10	104
		Street 18	0	198
		Street 19	11	245
		Street 20	0	64
		Street 21	44	321
		Street 22	8	53
		Street 23	6	26
		Street 24	4	213
		Street 25	0	161
		Street 26	0	22
		Street 1	0	89
		Street 2	0	31
		Street 3	2	42
		Street 4	3	110
		Street 5	6	58
		Street 6	128	200
		Street 7	9	94
		Street 8	15	91
		Street 9	6	69
		Street 10	0	64
		Street 11	25	326
		Street 12	84	134
	8:00	Street 13	5	34

	8:00	Street 14	6	21
		Street 15	3	16
		Street 16	23	143
		Street 17	15	66
		Street 18	0	34
		Street 19	8	76
		Street 20	4	30
		Street 21	19	184
		Street 22	4	25
		Street 23	22	12
		Street 24	4	125
		Street 25	1	34
		Street 26	0	10
	8:30	Street 1	7	81
		Street 2	2	29
		Street 3	5	49
		Street 4	6	52
		Street 5	1	28
		Street 6	106	140
		Street 7	8	122
		Street 8	9	108
		Street 9	12	64
		Street 10	3	72
		Street 11	17	99
		Street 12	70	93
		Street 13	3	39
		Street 14	2	36
		Street 15	2	22
		Street 16	14	163
		Street 17	14	30
		Street 18	2	20
		Street 19	5	48
		Street 20	3	20
		Street 21	10	88
		Street 22	3	29
		Street 23	6	21
		Street 24	1	42
		Street 25	0	14
		Street 26	0	39
		Street 1	1	69
		Street 2	1	35
		Street 3	2	37
		Street 4	4	52
		Street 5	2	15
		Street 6	81	101
		Street 7	14	82
		Street 8	8	109
		Street 9	18	37
		Street 10	2	55
		Street 11	14	69
		Street 12	54	67

	9:00	Street 13	4	22
		Street 14	1	16
		Street 15	2	12
		Street 16	12	162
		Street 17	26	31
		Street 18	2	17
		Street 19	11	31
		Street 20	1	5
		Street 21	8	58
		Street 22	1	20
		Street 23	4	13
		Street 24	1	56
		Street 25	2	15
		Street 26	1	21
	11:30	Street 1	5	36
		Street 2	2	26
		Street 3	4	28
		Street 4	6	42
		Street 5	3	20
		Street 6	34	65
		Street 7	25	100
		Street 8	9	38
		Street 9	8	22
		Street 10	2	41
		Street 11	6	95
		Street 12	21	43
		Street 13	6	36
		Street 14	2	32
		Street 15	3	15
		Street 16	12	57
		Street 17	14	38
		Street 18	0	152
		Street 19	15	35
		Street 20	1	18
		Street 21	3	75
		Street 22	1	14
		Street 23	11	20
		Street 24	9	51
		Street 25	1	22
		Street 26	0	34
		Street 1	2	66
		Street 2	1	18
		Street 3	1	52
		Street 4	2	54
		Street 5	1	13
		Street 6	37	60
		Street 7	14	62
		Street 8	17	41
		Street 9	9	36
		Street 10	1	77
		Street 11	21	164

	12:00	Street 12	24	40
		Street 13	6	24
		Street 14	2	21
		Street 15	2	11
		Street 16	10	62
		Street 17	11	26
		Street 18	3	27
		Street 19	6	44
		Street 20	2	26
		Street 21	6	76
		Street 22	1	37
		Street 23	6	49
		Street 24	5	64
		Street 25	1	32
		Street 26	1	18
	12:30	Street 1	1	25
		Street 2	2	15
		Street 3	2	35
		Street 4	9	93
		Street 5	3	9
		Street 6	35	55
		Street 7	7	50
		Street 8	5	48
		Street 9	6	21
		Street 10	1	52
		Street 11	16	225
		Street 12	23	36
		Street 13	2	17
		Street 14	3	12
		Street 15	1	3
		Street 16	8	72
		Street 17	12	44
		Street 18	3	21
		Street 19	3	51
		Street 20	2	14
		Street 21	8	79
		Street 22	5	9
		Street 23	5	22
		Street 24	8	30
		Street 25	5	38
		Street 26	0	16
		Street 1	1	40
		Street 2	2	21
		Street 3	2	54
		Street 4	6	74
		Street 5	2	10
		Street 6	37	75
		Street 7	9	48
		Street 8	13	46
		Street 9	3	28
		Street 10	0	82

	13:00	Street 11	23	113
		Street 12	25	50
		Street 13	5	16
		Street 14	6	11
		Street 15	2	9
		Street 16	20	59
		Street 17	18	26
		Street 18	1	33
		Street 19	4	32
		Street 20	6	10
		Street 21	5	41
		Street 22	0	18
		Street 23	8	32
		Street 24	2	46
		Street 25	2	22
		Street 26	0	31
	16:30	Street 1	4	40
		Street 2	4	21
		Street 3	1	131
		Street 4	8	74
		Street 5	2	21
		Street 6	46	123
		Street 7	6	98
		Street 8	17	105
		Street 9	13	16
		Street 10	3	81
		Street 11	17	96
		Street 12	31	81
		Street 13	1	23
		Street 14	3	11
		Street 15	1	10
		Street 16	32	91
		Street 17	4	58
		Street 18	0	17
		Street 19	12	79
		Street 20	3	25
		Street 21	3	221
		Street 22	0	17
		Street 23	6	61
		Street 24	5	178
		Street 25	5	59
		Street 26	3	22
		Street 1	4	162
		Street 2	4	46
		Street 3	1	52
		Street 4	8	47
		Street 5	4	23
		Street 6	136	144
		Street 7	10	131
		Street 8	18	79
		Street 9	12	108

	17:00	Street 10	0	77
		Street 11	11	143
		Street 12	90	95
		Street 13	8	55
		Street 14	6	52
		Street 15	2	23
		Street 16	27	119
		Street 17	53	75
		Street 18	0	21
		Street 19	9	58
		Street 20	4	22
		Street 21	9	275
		Street 22	0	15
		Street 23	15	45
		Street 24	2	282
		Street 25	0	17
		Street 26	1	27
	17:30	Street 1	7	111
		Street 2	2	55
		Street 3	6	68
		Street 4	2	70
		Street 5	8	15
		Street 6	93	156
		Street 7	21	301
		Street 8	22	59
		Street 9	11	58
		Street 10	5	100
		Street 11	18	187
		Street 12	62	103
		Street 13	16	116
		Street 14	18	78
		Street 15	9	56
		Street 16	33	89
		Street 17	28	87
		Street 18	2	11
		Street 19	8	29
		Street 20	2	27
		Street 21	25	192
		Street 22	3	32
		Street 23	11	64
		Street 24	10	85
		Street 25	0	9
		Street 26	4	41
		Street 1	3	60
		Street 2	3	30
		Street 3	1	33
		Street 4	3	140
		Street 5	1	15
		Street 6	88	105
		Street 7	10	117
		Street 8	7	46

	18:00	Street 9	8	37
		Street 10	3	48
		Street 11	16	162
		Street 12	59	70
		Street 13	4	29
		Street 14	2	22
		Street 15	3	20
		Street 16	10	69
		Street 17	39	92
		Street 18	2	7
		Street 19	17	321
		Street 20	2	12
		Street 21	13	123
		Street 22	2	30
		Street 23	5	33
		Street 24	2	31
		Street 25	1	14
		Street 26	0	14
Day 3	7:30	Street 1	5	221
		Street 2	2	66
		Street 3	2	109
		Street 4	2	199
		Street 5	11	50
		Street 6	50	234
		Street 7	30	476
		Street 8	8	102
		Street 9	7	189
		Street 10	1	162
		Street 11	8	245
		Street 12	34	155
		Street 13	21	169
		Street 14	19	63
		Street 15	11	55
		Street 16	11	152
		Street 17	10	97
		Street 18	1	178
		Street 19	15	269
		Street 20	1	71
		Street 21	47	279
		Street 22	10	69
		Street 23	3	78
		Street 24	3	302
		Street 25	2	201
		Street 26	1	33
		Street 1	5	79
		Street 2	2	44
		Street 3	0	47
		Street 4	0	98
		Street 5	1	46
		Street 6	45	205
		Street 7	19	121

	8:00	Street 8	9	154
		Street 9	8	36
		Street 10	1	70
		Street 11	11	289
		Street 12	30	136
		Street 13	11	63
		Street 14	6	36
		Street 15	9	22
		Street 16	13	102
		Street 17	5	58
		Street 18	1	46
		Street 19	4	84
		Street 20	2	24
		Street 21	20	123
		Street 22	2	33
		Street 23	1	21
		Street 24	0	112
		Street 25	6	55
		Street 26	2	14
	8:30	Street 1	5	74
		Street 2	4	27
		Street 3	1	44
		Street 4	1	47
		Street 5	1	32
		Street 6	74	139
		Street 7	9	129
		Street 8	12	110
		Street 9	7	55
		Street 10	2	65
		Street 11	17	102
		Street 12	50	92
		Street 13	6	36
		Street 14	2	26
		Street 15	3	21
		Street 16	17	165
		Street 17	10	68
		Street 18	0	26
		Street 19	4	55
		Street 20	0	29
		Street 21	29	78
		Street 22	1	36
		Street 23	2	21
		Street 24	1	56
		Street 25	2	31
		Street 26	1	30
		Street 1	4	54
		Street 2	2	49
		Street 3	3	37
		Street 4	4	59
		Street 5	2	12
		Street 6	46	108

	9:00	Street 7	10	81
		Street 8	16	123
		Street 9	9	19
		Street 10	4	54
		Street 11	8	88
		Street 12	31	72
		Street 13	5	45
		Street 14	2	26
		Street 15	2	31
		Street 16	23	184
		Street 17	19	50
		Street 18	0	31
		Street 19	9	30
		Street 20	0	21
		Street 21	2	35
		Street 22	2	21
		Street 23	2	13
		Street 24	3	71
		Street 25	2	40
		Street 26	1	22
	11:30	Street 1	2	28
		Street 2	1	37
		Street 3	2	22
		Street 4	0	41
		Street 5	2	16
		Street 6	31	60
		Street 7	17	108
		Street 8	8	47
		Street 9	8	16
		Street 10	5	34
		Street 11	11	100
		Street 12	20	40
		Street 13	11	88
		Street 14	6	59
		Street 15	8	61
		Street 16	13	70
		Street 17	16	33
		Street 18	1	44
		Street 19	11	32
		Street 20	3	17
		Street 21	11	369
		Street 22	4	34
		Street 23	13	25
		Street 24	11	69
		Street 25	3	26
		Street 26	0	24
		Street 1	2	59
		Street 2	2	20
		Street 3	1	51
		Street 4	7	57
		Street 5	2	15

	12:00	Street 6	36	70
		Street 7	8	69
		Street 8	11	50
		Street 9	6	33
		Street 10	3	76
		Street 11	14	169
		Street 12	24	47
		Street 13	9	56
		Street 14	3	36
		Street 15	1	26
		Street 16	16	74
		Street 17	9	25
		Street 18	2	52
		Street 19	5	48
		Street 20	3	33
		Street 21	9	145
		Street 22	4	29
		Street 23	3	61
		Street 24	6	54
		Street 25	0	18
		Street 26	3	11
	12:30	Street 1	1	22
		Street 2	1	14
		Street 3	3	36
		Street 4	6	92
		Street 5	1	11
		Street 6	1	60
		Street 7	11	57
		Street 8	12	45
		Street 9	11	18
		Street 10	1	54
		Street 11	24	198
		Street 12	0	39
		Street 13	4	23
		Street 14	3	21
		Street 15	2	15
		Street 16	17	67
		Street 17	14	39
		Street 18	2	42
		Street 19	4	49
		Street 20	1	9
		Street 21	6	204
		Street 22	1	45
		Street 23	7	32
		Street 24	6	66
		Street 25	2	33
		Street 26	2	16
		Street 1	3	48
		Street 2	2	27
		Street 3	2	53
		Street 4	7	80

	13:00	Street 5	2	13
		Street 6	45	77
		Street 7	7	54
		Street 8	8	36
		Street 9	7	32
		Street 10	2	78
		Street 11	6	109
		Street 12	30	52
		Street 13	1	22
		Street 14	2	18
		Street 15	2	10
		Street 16	12	55
		Street 17	21	22
		Street 18	0	17
		Street 19	4	11
		Street 20	8	9
		Street 21	9	9
		Street 22	2	12
		Street 23	7	22
		Street 24	3	96
		Street 25	3	37
		Street 26	2	29
	16:30	Street 1	3	55
		Street 2	3	9
		Street 3	2	64
		Street 4	7	24
		Street 5	2	5
		Street 6	118	113
		Street 7	8	112
		Street 8	20	61
		Street 9	6	21
		Street 10	3	66
		Street 11	17	14
		Street 12	79	79
		Street 13	2	18
		Street 14	3	10
		Street 15	1	3
		Street 16	31	98
		Street 17	36	54
		Street 18	0	15
		Street 19	10	27
		Street 20	4	24
		Street 21	22	126
		Street 22	3	36
		Street 23	10	69
		Street 24	3	102
		Street 25	4	96
		Street 26	1	12
		Street 1	5	41
		Street 2	1	24
		Street 3	2	54

	17:00	Street 4	5	76
		Street 5	5	21
		Street 6	119	115
		Street 7	14	106
		Street 8	13	48
		Street 9	9	28
		Street 10	4	80
		Street 11	22	112
		Street 12	79	76
		Street 13	9	58
		Street 14	6	26
		Street 15	6	22
		Street 16	20	73
		Street 17	55	56
		Street 18	1	29
		Street 19	6	84
		Street 20	5	18
		Street 21	21	206
		Street 22	4	21
		Street 23	12	54
		Street 24	3	211
		Street 25	0	112
		Street 26	2	20
	17:30	Street 1	6	152
		Street 2	1	64
		Street 3	4	52
		Street 4	4	51
		Street 5	4	22
		Street 6	103	130
		Street 7	19	140
		Street 8	18	103
		Street 9	15	127
		Street 10	6	78
		Street 11	23	241
		Street 12	68	87
		Street 13	11	112
		Street 14	3	69
		Street 15	6	61
		Street 16	27	154
		Street 17	31	69
		Street 18	3	44
		Street 19	12	51
		Street 20	0	29
		Street 21	9	258
		Street 22	1	40
		Street 23	11	21
		Street 24	8	96
		Street 25	1	58
		Street 26	0	33
		Street 1	2	97
		Street 2	2	51

	18:00	Street 3	2	64
		Street 4	3	77
		Street 5	2	29
		Street 6	79	160
		Street 7	12	317
		Street 8	7	59
		Street 9	9	63
		Street 10	1	97
		Street 11	17	298
		Street 12	52	106
		Street 13	5	201
		Street 14	2	156
		Street 15	3	103
		Street 16	10	89
		Street 17	36	106
		Street 18	1	9
		Street 19	14	337
		Street 20	1	8
		Street 21	5	132
		Street 22	5	29
		Street 23	12	29
		Street 24	5	58
		Street 25	3	37
		Street 26	0	17
Day 4	7:30	Street 1	4	245
		Street 2	2	73
		Street 3	1	121
		Street 4	5	200
		Street 5	12	44
		Street 6	55	250
		Street 7	18	441
		Street 8	9	116
		Street 9	5	178
		Street 10	2	181
		Street 11	18	213
		Street 12	37	166
		Street 13	11	366
		Street 14	6	319
		Street 15	9	266
		Street 16	14	173
		Street 17	12	99
		Street 18	0	177
		Street 19	6	214
		Street 20	3	66
		Street 21	47	384
		Street 22	13	46
		Street 23	4	112
		Street 24	1	236
		Street 25	2	119
		Street 26	1	19
		Street 1	4	69

	8:00	Street 2	1	36
		Street 3	2	48
		Street 4	1	96
		Street 5	2	54
		Street 6	65	187
		Street 7	25	130
		Street 8	12	99
		Street 9	13	36
		Street 10	3	71
		Street 11	13	333
		Street 12	43	125
		Street 13	13	87
		Street 14	10	76
		Street 15	6	59
		Street 16	18	149
		Street 17	7	69
		Street 18	0	51
		Street 19	3	99
		Street 20	2	31
		Street 21	18	187
		Street 22	2	31
		Street 23	2	36
		Street 24	0	156
		Street 25	1	69
		Street 26	1	6
	8:30	Street 1	7	77
		Street 2	3	32
		Street 3	1	41
		Street 4	3	48
		Street 5	1	18
		Street 6	65	132
		Street 7	20	132
		Street 8	13	111
		Street 9	16	59
		Street 10	0	62
		Street 11	15	74
		Street 12	44	87
		Street 13	11	100
		Street 14	9	78
		Street 15	8	59
		Street 16	18	166
		Street 17	8	54
		Street 18	2	33
		Street 19	5	75
		Street 20	0	27
		Street 21	29	77
		Street 22	1	33
		Street 23	1	31
		Street 24	2	61
		Street 25	3	55
		Street 26	2	10

	9:00	Street 1	7	65
		Street 2	3	54
		Street 3	1	35
		Street 4	2	44
		Street 5	5	19
		Street 6	45	102
		Street 7	12	64
		Street 8	13	124
		Street 9	8	56
		Street 10	2	53
		Street 11	4	84
		Street 12	30	67
		Street 13	5	45
		Street 14	5	41
		Street 15	2	26
		Street 16	18	186
		Street 17	7	66
		Street 18	1	27
		Street 19	4	39
		Street 20	0	16
		Street 21	5	59
		Street 22	3	29
		Street 23	3	45
		Street 24	2	77
		Street 25	2	87
		Street 26	2	17
	11:30	Street 1	4	39
		Street 2	1	37
		Street 3	0	27
		Street 4	3	49
		Street 5	2	10
		Street 6	27	57
		Street 7	25	119
		Street 8	7	46
		Street 9	5	22
		Street 10	0	40
		Street 11	12	215
		Street 12	17	37
		Street 13	11	85
		Street 14	9	66
		Street 15	5	36
		Street 16	10	83
		Street 17	15	57
		Street 18	0	125
		Street 19	18	27
		Street 20	1	22
		Street 21	9	336
		Street 22	4	56
		Street 23	13	59
		Street 24	3	158
		Street 25	2	29

		Street 26	3	21
	12:00	Street 1	2	57
		Street 2	1	17
		Street 3	1	46
		Street 4	6	48
		Street 5	4	20
		Street 6	35	61
		Street 7	5	74
		Street 8	7	48
		Street 9	9	39
		Street 10	3	68
		Street 11	21	369
		Street 12	23	41
		Street 13	1	55
		Street 14	0	34
		Street 15	2	22
		Street 16	9	71
		Street 17	12	40
		Street 18	1	34
		Street 19	6	48
		Street 20	5	16
		Street 21	4	144
		Street 22	4	44
		Street 23	10	23
		Street 24	1	111
		Street 25	6	37
		Street 26	1	14
	12:30	Street 1	2	27
		Street 2	1	18
		Street 3	1	37
		Street 4	5	94
		Street 5	2	16
		Street 6	2	58
		Street 7	12	59
		Street 8	8	44
		Street 9	6	21
		Street 10	2	55
		Street 11	29	214
		Street 12	1	38
		Street 13	6	31
		Street 14	5	36
		Street 15	2	24
		Street 16	13	65
		Street 17	13	61
		Street 18	2	8
		Street 19	8	56
		Street 20	2	22
		Street 21	12	206
		Street 22	2	41
		Street 23	7	31
		Street 24	2	69

		Street 25	4	22
		Street 26	0	19
	13:00	Street 1	2	41
		Street 2	1	29
		Street 3	0	52
		Street 4	6	86
		Street 5	3	11
		Street 6	51	79
		Street 7	5	48
		Street 8	2	39
		Street 9	18	33
		Street 10	2	78
		Street 11	7	166
		Street 12	33	52
		Street 13	3	20
		Street 14	2	22
		Street 15	2	26
		Street 16	1	59
		Street 17	17	50
		Street 18	3	31
		Street 19	4	33
		Street 20	4	15
		Street 21	6	95
		Street 22	0	30
		Street 23	10	46
		Street 24	2	55
		Street 25	3	35
		Street 26	0	14
	16:30	Street 1	2	43
		Street 2	3	26
		Street 3	1	52
		Street 4	8	77
		Street 5	1	26
		Street 6	111	113
		Street 7	9	100
		Street 8	19	52
		Street 9	10	26
		Street 10	1	77
		Street 11	14	120
		Street 12	73	75
		Street 13	4	37
		Street 14	1	30
		Street 15	1	22
		Street 16	29	78
		Street 17	66	77
		Street 18	0	10
		Street 19	16	94
		Street 20	2	17
		Street 21	31	198
		Street 22	5	31
		Street 23	3	16

		Street 24	1	63
		Street 25	2	112
		Street 26	1	11
	17:00	Street 1	3	149
		Street 2	2	70
		Street 3	3	51
		Street 4	3	53
		Street 5	4	22
		Street 6	121	139
		Street 7	11	139
		Street 8	12	90
		Street 9	6	102
		Street 10	2	76
		Street 11	29	248
		Street 12	80	92
		Street 13	8	67
		Street 14	3	39
		Street 15	4	45
		Street 16	17	136
		Street 17	21	59
		Street 18	1	17
		Street 19	3	61
		Street 20	4	25
		Street 21	15	166
		Street 22	2	33
		Street 23	14	22
		Street 24	9	335
		Street 25	2	97
		Street 26	0	6
	17:30	Street 1	6	109
		Street 2	2	59
		Street 3	5	68
		Street 4	9	81
		Street 5	2	17
		Street 6	88	168
		Street 7	16	299
		Street 8	12	54
		Street 9	15	88
		Street 10	3	101
		Street 11	11	296
		Street 12	59	111
		Street 13	12	206
		Street 14	9	112
		Street 15	4	106
		Street 16	17	80
		Street 17	19	44
		Street 18	2	20
		Street 19	14	34
		Street 20	3	36
		Street 21	15	125
		Street 22	3	42

		Street 23	2	29
		Street 24	4	112
		Street 25	1	22
		Street 26	0	12
	18:00	Street 1	4	55
		Street 2	2	50
		Street 3	1	28
		Street 4	2	141
		Street 5	1	18
		Street 6	100	114
		Street 7	11	99
		Street 8	8	46
		Street 9	9	36
		Street 10	2	41
		Street 11	16	488
		Street 12	67	75
		Street 13	8	22
		Street 14	3	18
		Street 15	5	11
		Street 16	10	70
		Street 17	28	60
		Street 18	0	15
		Street 19	22	291
		Street 20	0	21
		Street 21	6	112
		Street 22	0	42
		Street 23	6	14
		Street 24	3	34
		Street 25	1	36
		Street 26	0	34
Day 5	7:30	Street 1	9	280
		Street 2	4	80
		Street 3	2	106
		Street 4	2	187
		Street 5	13	51
		Street 6	54	238
		Street 7	21	470
		Street 8	6	111
		Street 9	17	231
		Street 10	1	159
		Street 11	14	243
		Street 12	35	159
		Street 13	15	217
		Street 14	6	189
		Street 15	6	155
		Street 16	9	165
		Street 17	15	125
		Street 18	1	200
		Street 19	15	216
		Street 20	2	57
		Street 21	47	337

		Street 22	8	44
		Street 23	7	66
		Street 24	2	289
		Street 25	5	156
		Street 26	1	31
	8:00	Street 1	5	84
		Street 2	0	39
		Street 3	1	48
		Street 4	0	116
		Street 5	4	49
		Street 6	51	199
		Street 7	19	101
		Street 8	11	105
		Street 9	16	65
		Street 10	3	73
		Street 11	15	336
		Street 12	34	132
		Street 13	14	36
		Street 14	8	33
		Street 15	9	26
		Street 16	16	156
		Street 17	8	54
		Street 18	0	51
		Street 19	8	81
		Street 20	1	21
		Street 21	21	118
		Street 22	6	21
		Street 23	2	25
		Street 24	3	97
		Street 25	2	36
		Street 26	1	12
	8:30	Street 1	2	80
		Street 2	1	22
		Street 3	2	48
		Street 4	0	50
		Street 5	2	22
		Street 6	78	131
		Street 7	14	121
		Street 8	12	107
		Street 9	10	64
		Street 10	2	71
		Street 11	10	91
		Street 12	52	87
		Street 13	9	36
		Street 14	5	26
		Street 15	6	11
		Street 16	18	161
		Street 17	6	74
		Street 18	0	31
		Street 19	3	59
		Street 20	3	36

		Street 21	39	70
		Street 22	3	30
		Street 23	5	21
		Street 24	2	69
		Street 25	2	54
		Street 26	2	14
	9:00	Street 1	4	66
		Street 2	2	39
		Street 3	2	35
		Street 4	0	40
		Street 5	2	16
		Street 6	42	103
		Street 7	14	87
		Street 8	15	120
		Street 9	5	33
		Street 10	3	52
		Street 11	4	89
		Street 12	25	69
		Street 13	8	55
		Street 14	7	26
		Street 15	5	22
		Street 16	23	179
		Street 17	11	63
		Street 18	2	29
		Street 19	4	33
		Street 20	1	22
		Street 21	8	38
		Street 22	4	26
		Street 23	5	44
		Street 24	0	55
		Street 25	6	74
		Street 26	1	15
	11:30	Street 1	3	27
		Street 2	0	24
		Street 3	1	25
		Street 4	1	58
		Street 5	1	11
		Street 6	34	78
		Street 7	15	104
		Street 8	7	36
		Street 9	9	26
		Street 10	2	37
		Street 11	18	223
		Street 12	23	51
		Street 13	12	87
		Street 14	6	59
		Street 15	9	55
		Street 16	9	57
		Street 17	24	47
		Street 18	1	104
		Street 19	6	41

		Street 20	2	31
		Street 21	14	441
		Street 22	2	21
		Street 23	9	56
		Street 24	5	120
		Street 25	5	38
		Street 26	2	21
	12:00	Street 1	0	61
		Street 2	3	19
		Street 3	0	47
		Street 4	8	47
		Street 5	2	17
		Street 6	32	60
		Street 7	4	70
		Street 8	6	45
		Street 9	5	45
		Street 10	1	70
		Street 11	22	364
		Street 12	22	40
		Street 13	1	41
		Street 14	0	36
		Street 15	0	22
		Street 16	8	67
		Street 17	7	40
		Street 18	0	51
		Street 19	2	45
		Street 20	2	17
		Street 21	9	225
		Street 22	6	30
		Street 23	2	33
		Street 24	2	112
		Street 25	0	29
		Street 26	1	13
	12:30	Street 1	0	21
		Street 2	0	16
		Street 3	1	35
		Street 4	7	99
		Street 5	1	8
		Street 6	1	65
		Street 7	9	62
		Street 8	13	45
		Street 9	3	18
		Street 10	0	53
		Street 11	36	241
		Street 12	0	43
		Street 13	5	22
		Street 14	3	15
		Street 15	2	11
		Street 16	18	67
		Street 17	8	66
		Street 18	1	14

		Street 19	4	68
		Street 20	4	30
		Street 21	12	231
		Street 22	2	11
		Street 23	2	29
		Street 24	6	64
		Street 25	2	31
		Street 26	0	24
	13:00	Street 1	2	39
		Street 2	1	22
		Street 3	1	55
		Street 4	8	79
		Street 5	4	11
		Street 6	49	76
		Street 7	6	52
		Street 8	1	41
		Street 9	8	36
		Street 10	2	82
		Street 11	15	159
		Street 12	32	51
		Street 13	3	30
		Street 14	2	32
		Street 15	2	16
		Street 16	2	62
		Street 17	14	51
		Street 18	1	31
		Street 19	4	46
		Street 20	5	24
		Street 21	11	117
		Street 22	3	15
		Street 23	6	44
		Street 24	4	54
		Street 25	3	20
		Street 26	0	18
	16:30	Street 1	3	44
		Street 2	2	27
		Street 3	1	52
		Street 4	6	80
		Street 5	4	23
		Street 6	118	108
		Street 7	10	103
		Street 8	20	51
		Street 9	11	25
		Street 10	2	78
		Street 11	12	99
		Street 12	78	72
		Street 13	8	59
		Street 14	5	36
		Street 15	4	44
		Street 16	29	75
		Street 17	59	88

		Street 18	1	17
		Street 19	14	88
		Street 20	4	21
		Street 21	22	216
		Street 22	1	16
		Street 23	11	11
		Street 24	7	47
		Street 25	2	69
		Street 26	0	9
	17:00	Street 1	3	144
		Street 2	1	51
		Street 3	2	53
		Street 4	6	48
		Street 5	4	26
		Street 6	110	137
		Street 7	17	143
		Street 8	11	84
		Street 9	9	126
		Street 10	4	78
		Street 11	29	215
		Street 12	73	91
		Street 13	11	67
		Street 14	6	45
		Street 15	9	36
		Street 16	15	125
		Street 17	24	59
		Street 18	0	23
		Street 19	3	67
		Street 20	4	25
		Street 21	17	258
		Street 22	3	18
		Street 23	20	21
		Street 24	3	266
		Street 25	1	66
		Street 26	2	8
	17:30	Street 1	4	114
		Street 2	1	57
		Street 3	8	64
		Street 4	2	76
		Street 5	5	13
		Street 6	91	162
		Street 7	14	311
		Street 8	20	59
		Street 9	2	87
		Street 10	7	96
		Street 11	14	263
		Street 12	60	108
		Street 13	9	159
		Street 14	4	112
		Street 15	5	36
		Street 16	30	88

		Street 17	15	55
		Street 18	3	21
		Street 19	11	22
		Street 20	2	26
		Street 21	11	116
		Street 22	2	36
		Street 23	13	36
		Street 24	5	196
		Street 25	3	24
		Street 26	1	10
	18:00	Street 1	1	59
		Street 2	2	37
		Street 3	3	31
		Street 4	1	117
		Street 5	2	10
		Street 6	84	100
		Street 7	10	107
		Street 8	5	49
		Street 9	12	36
		Street 10	1	46
		Street 11	12	502
		Street 12	56	66
		Street 13	4	63
		Street 14	2	37
		Street 15	2	22
		Street 16	8	72
		Street 17	33	57
		Street 18	0	18
		Street 19	21	306
		Street 20	3	14
		Street 21	10	98
		Street 22	1	24
		Street 23	6	22
		Street 24	5	42
		Street 25	6	10
		Street 26	0	33
Day 6	7:30	Street 1	1	87
		Street 2	1	14
		Street 3	4	24
		Street 4	3	95
		Street 5	1	12
		Street 6	33	153
		Street 7	9	134
		Street 8	7	31
		Street 9	6	56
		Street 10	2	35
		Street 11	8	60
		Street 12	21	101
		Street 13	5	88
		Street 14	1	65
		Street 15	5	56

	Street 16	11	47
	Street 17	10	31
	Street 18	2	7
	Street 19	3	24
	Street 20	6	9
	Street 21	11	68
	Street 22	2	19
	Street 23	4	9
	Street 24	3	38
	Street 25	4	18
	Street 26	1	8
	Street 1	4	92
	Street 2	2	19
	Street 3	4	43
	Street 4	9	74
	Street 5	9	17
	Street 6	52	125
	Street 7	8	128
	Street 8	5	34
	Street 9	15	69
	Street 10	2	64
	Street 11	14	59
	Street 12	34	82
8:00	Street 13	5	56
	Street 14	2	33
	Street 15	3	25
	Street 16	7	50
	Street 17	5	44
	Street 18	0	5
	Street 19	1	58
	Street 20	5	14
	Street 21	23	54
	Street 22	4	29
	Street 23	0	5
	Street 24	2	19
	Street 25	7	22
	Street 26	0	11
	Street 1	9	71
	Street 2	0	25
	Street 3	2	45
	Street 4	5	76
	Street 5	2	24
	Street 6	33	107
	Street 7	20	127
	Street 8	7	37
	Street 9	16	48
	Street 10	3	68
	Street 11	1	91
	Street 12	26	72
	Street 13	12	74
8:30	Street 14	11	56

	Street 15	8	63
	Street 16	11	55
	Street 17	12	29
	Street 18	0	3
	Street 19	0	46
	Street 20	7	12
	Street 21	7	89
	Street 22	3	17
	Street 23	5	16
	Street 24	2	19
	Street 25	2	5
	Street 26	5	12
9:00	Street 1	9	78
	Street 2		37
	Street 3	1	44
	Street 4		67
	Street 5		33
	Street 6	38	109
	Street 7	16	141
	Street 8	12	31
	Street 9	6	63
	Street 10	2	79
	Street 11	5	72
	Street 12	33	72
	Street 13		36
	Street 14	8	22
	Street 15	6	8
	Street 16	5	47
	Street 17	7	38
	Street 18	1	7
	Street 19	3	46
	Street 20	7	5
	Street 21	11	50
	Street 22	4	30
	Street 23	3	22
	Street 24	1	18
	Street 25	8	37
	Street 26	2	10
11:30	Street 1	1	63
	Street 2	2	15
	Street 3	3	55
	Street 4	7	62
	Street 5	0	11
	Street 6	61	52
	Street 7	7	112
	Street 8	10	39
	Street 9	8	44
	Street 10	1	82
	Street 11	14	78
	Street 12	26	34
	Street 13	2	45

	11:30	Street 14	1	22
		Street 15	1	19
		Street 16	14	58
		Street 17	10	62
		Street 18	3	10
		Street 19	10	72
		Street 20	3	14
		Street 21	16	59
		Street 22	3	37
		Street 23	7	22
		Street 24	4	26
		Street 25	1	30
		Street 26	0	15
	12:00	Street 1	2	51
		Street 2	2	31
		Street 3	4	47
		Street 4	6	66
		Street 5	2	8
		Street 6	29	75
		Street 7	13	67
		Street 8	14	46
		Street 9	12	46
		Street 10	5	71
		Street 11	5	55
		Street 12	19	49
		Street 13	11	27
		Street 14	9	21
		Street 15	6	22
		Street 16	19	59
		Street 17	7	54
		Street 18	2	15
		Street 19	8	68
		Street 20	0	20
		Street 21	8	67
		Street 22	4	23
		Street 23	10	39
		Street 24	5	29
		Street 25	2	15
		Street 26	0	17
		Street 1	4	46
		Street 2	2	16
		Street 3	2	39
		Street 4	3	93
		Street 5	1	14
		Street 6	42	47
		Street 7	3	65
		Street 8	9	43
		Street 9	13	54
		Street 10	3	57
		Street 11	16	61
		Street 12	28	31

	12:30	Street 13	1	33
		Street 14	3	31
		Street 15	1	24
		Street 16	12	63
		Street 17	12	52
		Street 18	0	8
		Street 19	9	60
		Street 20	7	11
		Street 21	14	71
		Street 22	0	28
		Street 23	4	26
		Street 24	3	17
		Street 25	3	15
		Street 26	1	17
	13:00	Street 1	4	60
		Street 2	1	33
		Street 3	1	51
		Street 4	1	68
		Street 5	2	11
		Street 6	65	79
		Street 7	12	87
		Street 8	6	58
		Street 9	18	59
		Street 10	2	76
		Street 11	14	62
		Street 12	43	52
		Street 13	8	51
		Street 14	6	26
		Street 15	5	13
		Street 16	8	73
		Street 17	7	39
		Street 18	0	5
		Street 19	6	75
		Street 20	3	6
		Street 21	9	54
		Street 22	0	51
		Street 23	3	17
		Street 24	0	21
		Street 25	2	16
		Street 26	0	14
		Street 1	5	82
		Street 2	1	55
		Street 3	3	26
		Street 4	7	73
		Street 5	7	9
		Street 6	94	101
		Street 7	24	59
		Street 8	8	30
		Street 9	9	48
		Street 10	5	40
		Street 11	20	111

	16:30	Street 12	62	67
		Street 13	18	33
		Street 14	15	12
		Street 15	6	15
		Street 16	11	45
		Street 17	19	32
		Street 18	1	12
		Street 19	5	58
		Street 20	2	18
		Street 21	10	64
		Street 22	4	19
		Street 23	3	11
		Street 24	5	26
		Street 25	5	21
		Street 26	11	11
	17:00	Street 1	2	64
		Street 2	0	91
		Street 3	3	25
		Street 4	2	59
		Street 5	3	14
		Street 6	72	151
		Street 7	14	22
		Street 8	2	27
		Street 9	17	36
		Street 10	6	38
		Street 11	8	237
		Street 12	48	100
		Street 13	9	10
		Street 14	6	11
		Street 15	9	8
		Street 16	4	39
		Street 17	11	26
		Street 18	2	18
		Street 19	3	33
		Street 20	1	10
		Street 21	8	71
		Street 22	2	45
		Street 23	6	16
		Street 24	2	11
		Street 25	2	29
		Street 26	20	18
		Street 1	6	62
		Street 2		41
		Street 3	5	30
		Street 4		51
		Street 5		11
		Street 6	99	181
		Street 7	12	98
		Street 8	16	45
		Street 9	4	37
		Street 10	5	44

	17:30	Street 11	9	93
		Street 12	55	120
		Street 13	11	36
		Street 14	5	31
		Street 15	6	16
		Street 16	11	38
		Street 17	11	41
		Street 18	4	7
		Street 19	5	25
		Street 20	0	14
		Street 21	7	41
		Street 22	2	23
		Street 23	2	9
		Street 24	3	12
		Street 25	3	22
		Street 26	10	9
	18:00	Street 1	3	46
		Street 2	2	33
		Street 3	3	15
		Street 4	2	84
		Street 5	0	16
		Street 6	37	129
		Street 7	12	69
		Street 8	8	42
		Street 9	6	26
		Street 10	4	24
		Street 11	10	57
		Street 12	24	85
		Street 13	11	42
		Street 14	11	36
		Street 15	9	22
		Street 16	12	62
		Street 17	22	43
		Street 18	2	6
		Street 19	6	53
		Street 20	1	10
		Street 21	0	45
		Street 22	2	33
		Street 23	3	5
		Street 24	1	20
		Street 25	1	16
		Street 26	5	4
**Day 7**		Street 1	1	76
		Street 2	1	19
		Street 3	4	25
		Street 4	4	88
		Street 5	5	17
		Street 6	43	322
		Street 7	9	127
		Street 8	11	26
		Street 9	8	66

	7:30	Street 10	4	37
		Street 11	9	66
		Street 12	29	215
		Street 13	3	100
		Street 14	3	54
		Street 15	5	39
		Street 16	17	40
		Street 17	8	27
		Street 18	1	6
		Street 19	4	21
		Street 20	7	11
		Street 21	9	74
		Street 22	5	19
		Street 23	4	8
		Street 24	4	37
		Street 25	3	10
		Street 26	3	6
	8:00	Street 1	5	76
		Street 2		24
		Street 3	3	44
		Street 4		70
		Street 5		21
		Street 6	60	129
		Street 7	9	120
		Street 8	3	37
		Street 9	11	69
		Street 10	1	69
		Street 11		49
		Street 12	30	85
		Street 13	4	67
		Street 14	2	66
		Street 15	2	25
		Street 16	6	56
		Street 17	3	33
		Street 18	0	7
		Street 19	2	46
		Street 20	8	16
		Street 21	25	50
		Street 22	3	22
		Street 23	2	6
		Street 24	1	10
		Street 25	5	28
		Street 26	1	8
		Street 1	1	68
		Street 2	1	17
		Street 3	2	44
		Street 4	2	68
		Street 5	5	19
		Street 6	46	118
		Street 7	4	130
		Street 8	1	39

	8:30	Street 9	9	45
		Street 10	1	65
		Street 11	16	84
		Street 12	31	78
		Street 13	2	68
		Street 14	1	35
		Street 15	3	21
		Street 16	0	60
		Street 17	9	48
		Street 18	1	2
		Street 19	1	49
		Street 20	16	10
		Street 21	14	99
		Street 22	2	15
		Street 23	4	20
		Street 24	2	15
		Street 25	2	7
		Street 26	4	6
	9:00	Street 1	2	86
		Street 2	1	33
		Street 3	2	48
		Street 4	7	73
		Street 5	13	34
		Street 6	48	106
		Street 7	6	134
		Street 8	4	6
		Street 9	15	67
		Street 10	3	71
		Street 11	6	66
		Street 12	32	70
		Street 13	0	88
		Street 14	1	48
		Street 15	0	44
		Street 16	6	7
		Street 17	12	29
		Street 18	2	12
		Street 19	2	63
		Street 20	12	8
		Street 21	19	61
		Street 22	2	24
		Street 23	3	18
		Street 24	3	17
		Street 25	11	24
		Street 26	3	11
		Street 1	13	73
		Street 2	1	14
		Street 3	0	58
		Street 4	4	66
		Street 5	1	9
		Street 6	35	51
		Street 7	16	127

	11:30	Street 8	6	40
		Street 9	22	62
		Street 10	2	85
		Street 11	9	64
		Street 12	23	33
		Street 13	11	59
		Street 14	6	36
		Street 15	5	22
		Street 16	10	60
		Street 17	18	55
		Street 18	3	7
		Street 19	14	79
		Street 20	12	13
		Street 21	23	63
		Street 22	4	41
		Street 23	10	31
		Street 24	2	31
		Street 25	6	29
		Street 26	1	17
	12:00	Street 1	0	64
		Street 2	1	38
		Street 3	0	48
		Street 4	2	62
		Street 5	1	6
		Street 6	38	72
		Street 7	3	60
		Street 8	8	43
		Street 9	5	58
		Street 10	2	72
		Street 11	12	51
		Street 12	25	47
		Street 13	1	23
		Street 14	0	12
		Street 15	2	16
		Street 16	12	64
		Street 17	10	50
		Street 18	1	10
		Street 19	6	49
		Street 20	2	24
		Street 21	10	65
		Street 22	5	19
		Street 23	12	36
		Street 24	3	22
		Street 25	4	18
		Street 26	0	12
		Street 1	1	41
		Street 2	0	20
		Street 3	3	35
		Street 4	5	94
		Street 5	3	8
		Street 6	24	48

	12:30	Street 7	7	61
		Street 8	12	46
		Street 9	6	45
		Street 10	3	53
		Street 11	18	72
		Street 12	15	32
		Street 13	2	36
		Street 14	2	15
		Street 15	1	11
		Street 16	18	67
		Street 17	22	34
		Street 18	1	6
		Street 19	5	61
		Street 20	4	9
		Street 21	19	59
		Street 22	1	24
		Street 23	6	22
		Street 24	2	18
		Street 25	2	11
		Street 26	2	15
	13:00	Street 1	5	54
		Street 2	1	40
		Street 3	3	43
		Street 4	2	71
		Street 5	4	18
		Street 6	37	76
		Street 7	3	81
		Street 8	7	44
		Street 9	9	36
		Street 10	2	65
		Street 11	10	60
		Street 12	25	51
		Street 13	0	66
		Street 14	1	63
		Street 15	0	25
		Street 16	10	65
		Street 17	10	36
		Street 18	0	6
		Street 19	7	77
		Street 20	5	10
		Street 21	12	71
		Street 22	2	61
		Street 23	2	18
		Street 24	1	19
		Street 25	5	19
		Street 26	1	14
		Street 1	5	79
		Street 2	2	58
		Street 3	2	30
		Street 4	2	69
		Street 5	4	11

	16:30	Street 6	67	110
		Street 7	14	63
		Street 8	7	33
		Street 9	11	56
		Street 10	1	44
		Street 11	99	94
		Street 12	44	74
		Street 13	13	12
		Street 14	5	11
		Street 15	8	13
		Street 16	9	49
		Street 17	21	27
		Street 18	3	10
		Street 19	3	56
		Street 20	1	21
		Street 21	15	69
		Street 22	3	23
		Street 23	2	10
		Street 24	1	22
		Street 25	3	21
		Street 26	12	13
	17:00	Street 1	8	69
		Street 2	0	78
		Street 3	1	34
		Street 4	9	62
		Street 5	6	17
		Street 6	122	148
		Street 7	6	28
		Street 8	0	25
		Street 9	19	52
		Street 10	3	50
		Street 11	213	206
		Street 12	81	98
		Street 13	1	20
		Street 14	2	26
		Street 15	1	13
		Street 16	0	36
		Street 17	15	29
		Street 18	1	12
		Street 19	1	37
		Street 20	1	15
		Street 21	16	84
		Street 22	1	35
		Street 23	4	11
		Street 24	5	26
		Street 25	5	16
		Street 26	19	10
		Street 1	4	64
		Street 2	2	30
		Street 3	0	26
		Street 4	7	49

	17:30	Street 5	8	15
		Street 6	118	173
		Street 7	28	113
		Street 8	8	42
		Street 9	13	47
		Street 10	1	38
		Street 11	88	89
		Street 12	78	115
		Street 13	22	6
		Street 14	22	2
		Street 15	15	5
		Street 16	13	62
		Street 17	12	36
		Street 18	4	5
		Street 19	4	36
		Street 20	2	10
		Street 21	11	36
		Street 22	0	29
		Street 23	11	16
		Street 24	3	15
		Street 25	5	9
		Street 26	11	21
	18:00	Street 1	3	50
		Street 2	1	29
		Street 3	0	19
		Street 4	3	79
		Street 5	3	21
		Street 6	97	136
		Street 7	16	78
		Street 8	3	38
		Street 9	26	33
		Street 10	2	27
		Street 11	79	61
		Street 12	65	90
		Street 13	8	41
		Street 14	6	25
		Street 15	2	29
		Street 16	5	56
		Street 17	22	39
		Street 18	1	9
		Street 19	4	59
		Street 20	6	10
		Street 21	8	39
		Street 22	2	24
		Street 23	3	6
		Street 24	4	16
		Street 25	6	13
		Street 26	8	8

Note: The data for each sample street included 5 weekdays and 2 weekend days for a total of 7 days, and the different types of physical activity were recorded in units of 0.5 h for each street for a total of 12 time points in the morning (7:30-9:00), midday (11:30-13:00) and evening (16:30-18:00)

The researchers used cameras to spot video the crowds passing within the sample streets and automatically extracted the number of physically active people and artificially classified them through Baidu's foot traffic statistics platform (https://cloud.baidu.com/product/body/num). The website interface is shown in the figure below

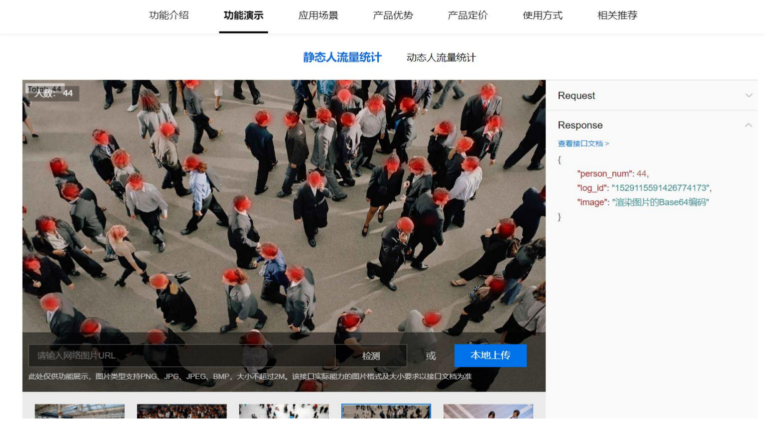

Supplement: Supplementary file 2 — Additional file 2. Measurement of physical activity indicators of users. [file 12889_2022_14533_MOESM2_ESM.pdf]
