# Supplementary material for: The effect of the street environment on two types of essential physical activity in industrial neighborhoods from the perspective of public health: a study from the Harbin low-income population health survey, China
Source: BMC Public Health. 2022 Nov 28;22:2201. doi: 10.1186/s12889-022-14533-7 (PMC9703667; doi:10.1186/s12889-022-14533-7)
Supplement: Supplementary file 3 — Additional file 3. Supplementary data for data analysis. [file 12889_2022_14533_MOESM3_ESM.docx]

1. **Self reported mental health of users**

| Descriptive statistics of all participants (N = 215, Harbin, China in 2021) | | | |
| --- | --- | --- | --- |
| Variables | Item | Count | Percentage (%) |
| Individual-level variables | | | |
| Gender | Male | 92 | 42.79 |
|  | Female | 123 | 57.21 |
| Age | 18-35 | 10 | 4.65 |
|  | 36-50 | 38 | 17.67 |
|  | 51-60 | 94 | 43.72 |
|  | 60 and above | 73 | 33.95 |
| Marital status | Single（Unmarried/Divorced/Widowed） | 50 | 23.26 |
|  | Married | 165 | 76.74 |
| Living arrangement | Alone | 19 | 8.84 |
|  | With spouse only | 80 | 37.21 |
|  | Two generations together | 34 | 15.81 |
|  | Three generations living under the same roof | 82 | 38.14 |
| Monthly income | Below ¥2000 | 104 | 48.37 |
|  | ¥2000-4999 | 79 | 36.74 |
|  | ¥5000-7999 | 19 | 8.84 |
|  | ¥8000 and above | 13 | 6.05 |
| Physical health-level variables | | | |
| Physical activity time | ＜30 mins per week | 64 | 29.77 |
|  | 30-149 mins per week | 100 | 46.51 |
|  | 150-299 mins per week | 27 | 12.56 |
|  | 300-449 mins per week | 11 | 5.12 |
|  | ≥450 mins per week | 13 | 6.05 |
| Physical activity frequency | None | 10 | 4.65 |
|  | Once a week | 55 | 25.58 |
|  | 2-4 times a week | 78 | 36.28 |
|  | 5-7 times a week | 62 | 28.84 |
|  | More than 7 times a week | 10 | 4.65 |
| Physical health status | Very good | 54 | 25.12 |
|  | Good | 57 | 26.51 |
|  | Medium | 53 | 24.65 |
|  | Not good | 51 | 23.72 |
| Depressed mood | None | 8 | 3.72 |
|  | Occasionally | 21 | 9.77 |
|  | Often | 120 | 55.81 |
|  | Invariably | 66 | 30.7 |
| Chronic disease | Non-existent | 33 | 15.32 |
|  | Existence | 182 | 84.65 |

Note：Self-reported data were obtained by means of questionnaires and field visits, included basic personal information and self-assessment of health status.

1. **Pearson correlation analysis between the linear spatial street environment and the two types of necessary physical activity of the inhabitants of an industrial neighbourhood street**

|  | life-type physical activity | traffic-type physical activity |
| --- | --- | --- |
| D1a | 0.260^**^ | -0.087^**^ |
| D1b | 0.314^**^ | 0.114^**^ |
| D1c | -0.072^**^ | -0.061^**^ |
| D1d | -0.117^**^ | 0.112^**^ |
| D1e | 0.071^**^ | 0.078^**^ |
| D1f | -0.327^**^ | -0.043^**^ |
| D2 | 0.146^**^ | -0.022 |
| D3a | -0.135^**^ | 0.055^**^ |
| D3b | -0.017 | 0.085^**^ |
| D3c | -0.021 | 0.199^**^ |
| D3d | -0.140^**^ | 0.040 |
| D3e | -0.148^**^ | -0.035 |
| D3f | -0.138^**^ | -0.061^**^ |
| D3g | -0.208^**^ | -0.094^**^ |
| D3h | 0.008 | 0.053^*^ |
| D3i | -0.095^**^ | -0.045^*^ |
| D3j | -0.173^**^ | -0.052^*^ |
| D3k | 0.000 | 0.051^*^ |
| D3l | 0.169^**^ | 0.040 |
| DA1 | -0.060 | -0.173^**^ |
| DA2 | -0.092^**^ | 0.144^**^ |
| DA3 | -0.147^**^ | 0.191^**^ |
| DTT1 | -0.124^**^ | 0.125^**^ |
| DTT2 | -0.133^**^ | 0.102^**^ |

Pearson correlation coefficient values and significance values, *p < .05, **p < .01, statistics significant at p < .05 are displayed in bold.

Note：Table shows the results of the Pearson correlation between the linear spatial street environment and the two types of necessary physical activity of the inhabitants of an industrial neighbourhood street.

1. **Classification of street environment indicators of necessary physical activity.**

The different effects of each street environment indicator on the two types of necessary physical activity were further analyzed. After removing the street environment indicators that were not correlated in the bivariate correlation analysis, the main components of the street environment indicators that influenced the different essential types of physical activity were further clarified based on the principal component analysis method.

1. Life-style physical activity principal component analysis test sample

| **KMO and Bartlett's Test** | | |
| --- | --- | --- |
| Kaiser-Meyer-Olkin Measure of Sampling Adequacy. | | .635 |
| Bartlett's Test of Sphericity | Approx. Chi-Square | 72072.047 |
|  | df | 171 |
|  | Sig. | .000 |


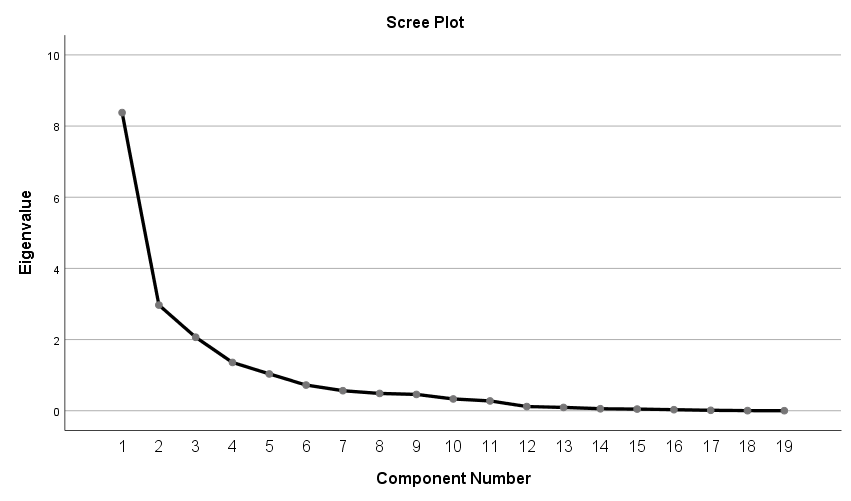


1. Traffic-style physical activity principal component analysis test sample

| **KMO and Bartlett's Test** | | |
| --- | --- | --- |
| Kaiser-Meyer-Olkin Measure of Sampling Adequacy. | | .586 |
| Bartlett's Test of Sphericity | Approx. Chi-Square | 63630.897 |
|  | df | 171 |
|  | Sig. | .000 |


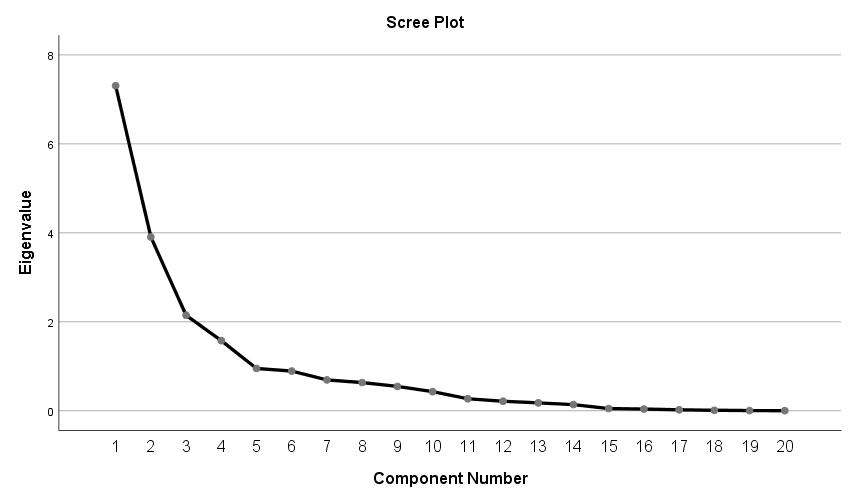


Note：In both principal component analyses, the KMO of the test sample was greater than 0.5, and the Sig of Bartlett's spherical test was equal to 0.000 < 1%. Based on the comparison of the data, it can be concluded that the data in the paper are suitable for principal component analysis.

1. **Construction of regression models for the street environment indicators of necessary physical activity.**
2. Results of multiple linear regression analysis of life-style physical activity

| **Model Summary^b^** | | | | | |
| --- | --- | --- | --- | --- | --- |
| Model | R | R Square | Adjusted R Square | Std. Error of the Estimate | Durbin-Watson |
| 1 | .721^a^ | .521 | .516 | 12.01748 | 1.721 |
| a. Predictors: (Constant), DTT2, D1f, D1b, DA3, D2, D1c, D3i, D1e, DA2, D3e, D3g, D1a, DTT1, D3d, D1d, D3l, D3a, D3f, D3j | | | | | |
| b. Dependent Variable: life-type physical activity | | | | | |

| **Coefficients^a^** | | | | | | | | |
| --- | --- | --- | --- | --- | --- | --- | --- | --- |
| Model | | Unstandardized Coefficients | | Standardized Coefficients | t | Sig. | Collinearity Statistics | |
|  |  | B | Std. Error | Beta |  |  | Tolerance | VIF |
|  | Number of bus stations  (D3b) | -5.763 | .566 | -.485 | -10.175 | .000 | .100 | 9.990 |
|  | Green space accessibility (DA3) | -.016 | .001 | -.564 | -13.902 | .000 | .138 | 7.250 |
|  | Density of production type stores along the street  (D1c) | -3.115 | .207 | -.304 | -15.021 | .000 | .555 | 1.803 |
|  | Diversity of commercial businesses along the street  (D2) | 41.878 | 2.743 | .594 | 15.266 | .000 | .150 | 6.670 |
|  | Greening density  (D1f) | -.500 | .042 | -.468 | -11.987 | .000 | .149 | 6.718 |
| a. Dependent Variable: life-type physical activity | | | | | | | | |

1. Results of multiple linear regression analysis of traffic-style physical activity

| **Model Summary^b^** | | | | | |
| --- | --- | --- | --- | --- | --- |
| Model | R | R Square | Adjusted R Square | Std. Error of the Estimate | Durbin-Watson |
| 1 | .563^a^ | .317 | .310 | 54.55907 | 1.448 |
| a. Predictors: (Constant), DTT2, D3c, D1b, D2f, D3h, DA1, D3i, D1c, DA2, D2e, DA3, D1a, D3b, D3g, DTT1, D2d, D3f, D3a, D3j, D3k | | | | | |
| b. Dependent Variable: traffic-type physical | | | | | |

| **Coefficients^a^** | | | | | | | | |
| --- | --- | --- | --- | --- | --- | --- | --- | --- |
| Model | | Unstandardized Coefficients | | Standardized Coefficients | t | Sig. | Collinearity Statistics | |
|  |  | B | Std. Error | Beta |  |  | Tolerance | VIF |
|  | Density of life type stores along the street  （D1a） | -5.491 | .402 | -.687 | -13.675 | .000 | .126 | 7.966 |
|  | Community accessibility  （DA2） | .200 | .097 | .112 | 2.059 | .040 | .107 | 9.386 |
|  | Number of road intersections  （D3b） | 10.122 | 3.892 | .123 | 2.601 | .009 | .141 | 7.094 |
|  | Greening density  （D2f） | -2.234 | .174 | -.550 | -12.808 | .000 | .172 | 5.808 |
|  | Plant accessibility  （DA1） | -.106 | .007 | -.815 | -14.958 | .000 | .107 | 9.364 |
| a. Dependent Variable: life-type physical activity | | | | | | | | |

Note：In this paper, street environment indicators were used as independent variables, the number of people undertaking two types of essential physical activity during the research period was used as dependent variables, and two multiple linear regression models were constructed using SPSS software. The coefficients of the influencing factors were ranked, and the top 5 street environment indicators in terms of degree of influence were extracted. The VIFs of the 2 models were <10, and there was no significant covariance problem.
